# Screening and identification of fungal species associated with early-stage deterioration of natural rubber

**DOI:** 10.3897/mycokeys.134.195079

**Published:** 2026-06-16

**Authors:** Rui-Fang Xu, Samantha C. Karunarathna, Kevin D. Hyde, Chayanard Phukhamsakda, Sylvie Rapior, Dong-Qin Dai, Mei-Mei Wang, Sajad Ali, Jing-Ya Yang, Pattana Kakumyan, Saowaluck Tibpromma

**Affiliations:** 1 Center for Yunnan Plateau Biological Resources Protection and Utilization, College of Biology and Food Engineering, Qujing Normal University, Qujing 655099, China College of Science, King Faisal University Al-Ahsa Saudi Arabia; 2 Key Laboratory of Yunnan Provincial Department of Education of the Deep-Time Evolution on Biodiversity from the Origin of the Pearl River, Qujing Normal University, Qujing, Yunnan Province, 655011, China School of Science, Mae Fah Luang University Chiang Rai Thailand; 3 School of Science, Mae Fah Luang University, Chiang Rai, 57100, Thailand Center of Excellence in Fungal Research, Mae Fah Luang University Chiang Rai Thailand; 4 Center of Excellence in Fungal Research, Mae Fah Luang University, Chiang Rai, 57100, Thailand Advanced Microbial Metabolites and Phytochemical Technology, Mae Fah Luang University Chiang Rai Thailand; 5 Department Microbial Drugs, Helmholtz Centre for Infection Research (HZI), Inhoffenstrasse 7, 38124, Braunschweig, Germany Microbial Products and Innovations Research Group, Mae Fah Luang University Chiang Rai Thailand; 6 CEFE, Univ Montpellier, CNRS, EPHE, IRD, Laboratory of Botany, Phytochemistry and Mycology, Faculty of Pharmacy, Montpellier, France College of Biology and Food Engineering, Qujing Normal University Qujing China; 7 Department of Biological Sciences, College of Science, King Faisal University, Al-Ahsa 31982, Saudi Arabia Key Laboratory of Yunnan Provincial Department of Education of the Deep-Time Evolution on Biodiversity from the Origin of the Pearl River, Qujing Normal University Qujing China; 8 College of Agronomy and Biological Science, Yuxi Normal University, Yuxi, Yunnan 653100, China Helmholtz Centre for Infection Research (HZI) Braunschweig Germany; 9 Microbial Products and Innovations Research Group, Mae Fah Luang University, Chiang Rai, 57100, Thailand College of Agronomy and Biological Science, Yuxi Normal University Yuxi China; 10 Advanced Microbial Metabolites and Phytochemical Technology, Mae Fah Luang University, Chiang Rai 57100 Thailand CEFE, Univ Montpellier Montpellier France

**Keywords:** Enzymatic screening, fungi, multilocus phylogeny, surface modification, taxonomy

## Abstract

Natural rubber is an important biopolymer utilized in more than 4,000 commercial products. Nevertheless, the accumulation of waste rubber poses a significant environmental challenge due to its resistance to natural degradation. The present study sought to isolate and identify fungi from discarded rubber materials and assess their potential involvement in the early-stage surface deterioration of natural rubber. An initial screening of 69 fungal strains for extracellular enzyme production, including esterases, lipases, proteases, and laccases, was conducted. Following preliminary qualitative assays, 36 enzyme-producing isolates were further assessed for their capacity to modify natural rubber using rubber disc assays. Four isolates (L-12A, L-25, L-33, and T-21) showed measurable surface alterations associated with early-stage deterioration, as evidenced by limited mass loss, positive Schiff’s reagent staining, and surface alterations observed through scanning electron microscopy (SEM). After 2 months of incubation, rubber mass loss ranged from 0.77% ± 0.27% to 1.24% ± 0.25%. Schiff’s reagent staining indicated oxidative modification of the rubber surface, and SEM analysis revealed surface cracking, erosion-like features, and fungal colonization. Combined morphological characteristics and multilocus phylogenetic analyses identified these strains as *Neocosmospora bostrycoides* (L-12A), *Neocosmospora* sp. (T-21), *Paracremonium laticis* (L-25), and *Schizophyllum commune* (L-33). Descriptions, illustrations, and phylogenetic analyses of the four species are presented. This study provides preliminary evidence that these fungi may contribute to early-stage surface deterioration and oxidative modification of natural rubber.

## Introduction

Natural rubber is an elastomer primarily composed of *cis*-1,4-polyisoprene, which is naturally produced in the latex of the rubber tree, *Hevea brasiliensis* (Andler et al. 2022). Its exceptional flexibility and extensibility make it suitable for a wide range of everyday products (Basik et al. 2021a). Global rubber consumption continues to rise, driven by urbanization, economic growth, and population growth (Zhao et al. 2024). Global natural rubber production exceeds 14 million tons annually (Rubber Research Institute, Chinese Academy of Tropical Agricultural Sciences 2026). Concurrently, the automotive industry contributes nearly 1 billion waste tires annually (Zaki et al. 2025). Because of its unique structure, rubber does not break down easily and can remain in the environment for decades, thereby contributing to the increasing accumulation of rubber waste (Fazli and Rodrigue 2020; El-Wafai et al. 2023). Consequently, rubber waste has been recognized as one of the most significant components of solid waste worldwide (Fazli and Rodrigue 2020).

Traditional rubber waste management primarily relies on open dumping, landfilling, and incineration, all of which pose significant environmental risks (Pathiraja et al. 2025). Landfilling can release toxic substances and heavy metals into groundwater, whereas incineration can emit harmful pollutants, including carbon dioxide, sulfur dioxide, and fine particulate matter (PM_2.5_) (Puello-Mendez et al. 2022). These processes collectively contribute to air, soil, and groundwater pollution, which has a significant impact on local ecosystems. Additionally, they have the potential to cause human diseases (Basik et al. 2021a; Leong et al. 2023; Krasaesin et al. 2025). Considering these challenges, it is essential to develop environmentally friendly solutions for the disposal of rubber waste. Recent studies have found that carbonized waste tire rubber exhibits a rough surface and a uniform distribution of fine particles, making it suitable for the development of novel battery electrodes (Xiao et al. 2025). In addition to the above-mentioned treatment methods, microbial-assisted deterioration and biotransformation have been considered environmentally friendly alternatives for managing waste polymers. This process relies on microorganisms to decompose complex organic compounds in solid waste into simpler molecules and biomass, ultimately converting them into water, carbon dioxide, or methane (Nayanashree and Thippeswamy 2015a; Basik et al. 2021a; Chittella et al. 2021; Roy and Chakraborty 2024).

Research into the microbial degradation of rubber has been ongoing since 1914 (Söhngen and Fol 1914; Rose and Steinbüchel 2005). Numerous microorganisms have been reported to interact with, colonize, or modify rubber-containing materials (Joseph et al. 2022; Cui et al. 2023; El-Wafai et al. 2023; Prakash et al. 2024; Guajardo-Flores et al. 2025). These degraders are divided into two distinct groups based on their mechanisms of interaction with rubber substrates (Chia et al. 2014; Kasai 2020). The first group comprises natural rubber-utilizing strains that form visible clear zones, or transparent halos, around colonies when cultured on opaque rubber agar plates. These surface-clearing activities can be visually observed (Chia et al. 2014; Basik et al. 2021a; Cui et al. 2023). The second group includes rubber-degrading microorganisms that do not produce clear zones on latex-containing agar. This group of microorganisms directly attacks the rubber matrix, forming biofilms that begin degradation at the cell surface (Chia et al. 2014; Cui et al. 2023).

Rubber-degrading fungi have been identified from various genera, including *Alternaria* (Bosco et al. 2018), *Aspergillus* (Mohamed et al. 2017), *Fusarium* (Borel et al. 1982), *Penicillium* (Mohamed et al. 2017), *Monascus* (Schade 1937), *Mucor* (Atagana et al. 1999), and *Resinicium* (Bredberg et al. 2002), showing reported associations with surface alteration or deterioration of natural rubber, vulcanized rubber, rubber tires, and polyisoprene (Table 1) (Basik et al. 2021a; Joseph et al. 2022; Cui et al. 2023).

**Table 1.** Rubber-degrading fungi.

Fungal species	Substrate/source	Growth/surface alteration material	References
*Alternaria alternata*	Soil and rubber surface	Natural rubber	Bosco et al. 2018
	Surface of non-sterile natural rubber	Natural rubber sheets	Mollea and Bosco 2020
*Aspergillus flavus*	Soil	Natural rubber latex from *Ficus elastica*	Ismail et al. 2013
*Aspergillus niger*	Leaves and latex of *Calotropis procera*	Natural rubber latex	Mohamed et al. 2017
*Aspergillus oryzae*	Landfills, wastewater, and activated sludge	Rubber powder waste	El-Wafai et al. 2023
*Aspergillus terreus*	Soil	Natural rubber latex from *Ficus elastica*	Ismail et al. 2013
*Ceriporiopsis subvermispora*	Cultured from a basidiome	Vulcanized natural rubber sheets	Sato et al. 2004
*Cladosporium cladosporioides*	Soils and deteriorated tires	Rubber particles	Borel et al. 1982
*Fusarium solani* (synonym *Neocosmospora solani*)	Soils and deteriorated tires	Rubber particles	Borel et al. 1982
*Mucor racemosus*	Natural rubber waste serum	Capable of utilizing natural rubber waste serum	Atagana et al. 1999
*Mucor* sp.	Natural rubber waste serum	Capable of utilizing natural rubber waste serum	Atagana et al. 1999
*Thermothelomyces thermophiles* (synonym *Myceliophthora thermophila*)	Soil	Natural rubber latex from *Ficus elastica*	Ismail et al. 2013
*Monascus olei* (synonym *Monascus purpureus*)	Rice grains (Koji*)	Unvulcanized rubber samples	Schade 1937
*Monascus ruber*	Crude rubber	Unvulcanized rubber samples	Schade 1937
*Purpureocillium lilacinum* (synonym *Paecilomyces lilacinus*)	Soils and deteriorated tires	Rubber particles	Borel et al. 1982
*Paecilomyces variotii*	Isolated from a hot spring	Synthetic rubber	Shah et al. 2013
*Penicillium chrysogenum*	Leaves and latex of *Calotropis procera*	Natural rubber latex	Mohamed et al. 2017
	Natural rubber latex sheet (for burial in the ground)	Natural rubber discs	Nayanashree and Thippeswamy 2015b
	Surface of non-sterile natural rubber	Natural rubber sheets	Mollea and Bosco 2020
*Talaromyces variabilis* (synonym *Penicillium variabile*)	Deteriorated natural rubber after soil burial	Natural rubber smoked sheet	Williams 1982
*Phlebia radiata*	White rot fungi	Natural rubber	Singh et al. 2017
*Phlebiopsis* sp.	Lignocellulolytic fungi	Tire powder	Enciso-Sáenz et al. 2025
*Juxtiphoma eupyrena* (synonym *Phoma eupyrena*)	Soils and deteriorated tires	Rubber particles	Borel et al. 1982
*Pleurotus ostreatus*	White rot fungi	Rubber particles	Andler et al. 2021
		Waste tire rubber	Puello-Mendez et al. 2022
*Resinicium bicolor*	White rot fungi	Cryo-ground tire rubber (detoxification)	Bredberg et al. 2002
*Rhodotorula mucilaginosa*	Soil and rubber surface	Unvulcanized natural rubber	Bosco et al. 2018
*Earliella scabrosa* (synonym *Trametes sanguinea*)	Cultured from a basidiome	Tire powder	Enciso-Sáenz et al. 2025
*Trametes versicolor*	Cultured from a basidiome	Rubber particles	Andler et al. 2021

(*Koji is a Japanese culinary staple consisting of steamed rice or soybeans inoculated with the fungus *Aspergillus oryzae*).

Previous studies have shown that microbial interactions with rubber materials may involve extracellular oxidative enzymes that modify polyisoprene surfaces (Joseph et al. 2022). In bacteria, several rubber oxygenases, including rubber oxygenase A (RoxA), rubber oxygenase B (RoxB), and latex clearing protein (Lcp), have been shown to catalyze the oxidative cleavage of polyisoprene chains (Basik et al. 2021a). In fungi, oxidative enzymes such as laccases and peroxidases have also been suggested to contribute to rubber surface alteration through non-specific oxidation reactions (Nayanashree and Thippeswamy 2015a, 2015b; Andler et al. 2021; Basik et al. 2021a). However, the mechanisms underlying fungal-mediated rubber deterioration remain poorly understood.

Although research on rubber biodegradation has increased, most studies have focused on bacterial degraders and the bacterial rubber oxygenases RoxA, RoxB, and Lcp (Basik et al. 2021a). In contrast, the diversity of fungi involved in natural rubber degradation, as well as the mechanisms underlying fungal-mediated degradation, remains insufficiently characterized. Existing studies have primarily documented fungal degradation within a limited number of genera, leaving many fungal taxa associated with deteriorated rubber materials unexamined (El-Wafai et al. 2023). Additionally, the roles of fungal extracellular enzymes, particularly oxidative enzymes such as laccases and peroxidases, in modifying natural rubber are not well understood. Further research is required to broaden the recognized diversity of rubber-associated fungi and to clarify their potential roles in the early-stage surface deterioration of rubber materials.

The objective of this study was to isolate and identify fungi associated with discarded natural latex materials and preliminarily evaluate their potential involvement in early-stage surface deterioration of natural rubber. A multi-stage screening strategy was employed, including preliminary extracellular enzyme screening followed by surface alteration assessment using weight-loss analysis, Schiff’s reagent staining, and scanning electron microscopy (SEM). Positive fungal isolates were identified through a combination of morphological characterization and multilocus phylogenetic analysis. This study expands the known diversity of fungi associated with deteriorated rubber materials and provides preliminary evidence for the involvement of *Neocosmospora*, *Paracremonium*, and *Schizophyllum* in natural rubber surface alteration processes.

## Materials and methods

### Sample collection

Latex samples were collected from tree trunks, the ground, and a container located near the rubber trees during the production phase, as well as from aged or deteriorated rubber latex materials collected from farmers’ rubber collection workshops in China and Thailand. Important collection details, including the collecting site and date, habitat, and substrates, were recorded in accordance with the instructions in Rathnayaka et al. (2025). After collection, the samples were taken to the laboratory in sealed plastic bags for further experiments.

### Isolation fungi

Two isolation methods were used. In the first method, direct culturing, fragments of aged latex exhibiting mycelial masses or fungal strains were aseptically excised, placed on potato dextrose agar (PDA) medium under laminar flow conditions, and incubated at 25 °C until hyphal growth was observed (Xu et al. 2024). For the dilution plating method, latex samples were washed with sterile water to remove surface impurities, air-dried, and had the outer layers trimmed. A total of 10 g of prepared fragments were transferred to an Erlenmeyer flask containing 90 mL of sterile water and glass beads. The flask was agitated for 30 min. Serial dilutions (10^-2^ to 10^-9^) were prepared, and 0.1 mL aliquots from selected dilutions were spread onto PDA plates. Plates were incubated at 25 °C (Davis et al. 2005). Pure isolates were obtained by subculturing fungal strains after 3–5 days of incubation.

### Screening for enzyme production by isolated fungi

All isolated strains were screened for esterase, protease, lipase, and laccase production. For each enzyme assay, a single experimental group was used for every strain. The enzymatic assays were conducted as preliminary qualitative screening tests intended for the rapid assessment of extracellular enzymatic activities.

### Esterase production

Esterase production was evaluated on a medium containing (g/L): peptone (10), yeast extract (1), calcium chloride (0.05), agar (15), and 1% Tween 20 (Castro et al. 1992; El-Morsy 2017; Ren et al. 2021). One- to two-month-old fungal mycelial plugs (5 mm) were placed in the center of the esterase medium (9 cm diameter Petri dish) and incubated at 28 °C for 7 days. The control group was not inoculated; the medium was supplemented with 10 mg of esterase powder. A positive reaction was indicated by a white halo around the strain due to calcium salt precipitation induced by the addition of 10 mg of esterase powder to the medium. Relative enzyme activity (RA) was calculated as the ratio of the enzyme zone diameter to the colony diameter (Boonrung et al. 2023):

RA=enzyme zone diameter / colony diameter

### Protease production

Protease production was evaluated using a casein-based medium containing (g/L): casein (10), peptone (5), yeast extract (2.5), KH_2_PO_4_ (0.3), MgSO_4_ (0.5), NaCl (1), and agar (15) (Mohamed et al. 2023). One- to two-month-old fungal mycelial plugs (5 mm) were placed in the center of the protease medium (9 cm diameter Petri dish) and incubated at 28 °C for 7 days. The control group was not inoculated; the medium was supplemented with 10 μL of protease. A clear zone around the colony indicated protease activity. RA was calculated as described above.

### Lipase production

Lipase production was evaluated using a solid medium with the following composition (g/L): peptone (10), NaCl (5), CaCl_2_·H_2_O (0.1), agar (15), and Tween 80 at a concentration of 1% (v/v) (Pham et al. 2021). One- to two-month-old fungal mycelial plugs (5 mm) were placed in the middle of the lipase medium (9 cm diameter Petri dish) and incubated at 28 °C for 7 days. The control group was not inoculated with the strain; 10 mg of lipase powder was added to the medium. The formation of a white precipitate (calcium oleate) indicated lipase activity. Lipase hydrolyzes Tween 80, releasing oleic acid. The free oleic acid subsequently binds with calcium ions in the medium to form insoluble calcium oleate, resulting in a white precipitate around the microbial colonies (Lanka and Latha 2015). The lipase production capabilities were quantified as RA, as described above.

### Laccase production

Laccase production was assessed using a solid medium with the following composition (g/L): peptone (3.0), glucose (10.0), KH_2_PO_4_ (0.6), K_2_HPO_4_ (0.4), MgSO_4_ (0.5), MnSO_4_ (0.05), FeSO_4_ (0.0005), ZnSO_4_ (0.001), and agar (20.0). The medium was supplemented with 0.02% (v/v) guaiacol as a chromogenic substrate (Nayanashree and Thippeswamy 2015b). One- to two-month-old fungal mycelial plugs (5 mm) were placed in the center of the laccase medium (9 cm diameter Petri dish) and incubated at 28 °C for 7 days. The control group was not inoculated; 10 mg of laccase powder was added to the medium. Positive laccase activity was indicated by reddish-brown zones around the colonies (Nayanashree and Thippeswamy 2015b).

These assays were intended only for preliminary isolate selection and do not represent quantitative measurements of enzymatic activity or direct evidence of rubber polymer degradation. A clustered heatmap illustrating the relative activity of esterase, lipase, and protease for the 36 positive strains was generated using the online platform Chiplot (https://www.chiplot.online/).

### Screening of fungal isolates using natural rubber substrates

Natural rubber blocks (diameter: 6.35 cm, thickness: 1.9 cm) were manually sectioned into thin discs (2–3 mm thickness), washed, air-dried, weighed, and sterilized. Fungal strains with a 5 mm, 2-month-old mycelial plug were inoculated onto PDA plates, and a sterilized natural rubber disc was placed on each plate. Plates were incubated at 25 °C for 2 months, with three biological replicates per strain and a non-inoculated control.

After incubation, the discs were collected, washed, dried in a vacuum-drying oven at 40 °C for 48 h, and reweighed (Ren et al. 2021):

M (%) = M1–M2 / M1×100%,

where M (%) represents the percentage mass loss, M1 (g) is the initial mass of the natural rubber disc before incubation, and M2 (g) is the final mass after incubation. Triplicate measurements were averaged to minimize experimental variability. Mass loss was used only as a preliminary indicator of potential surface-associated deterioration.

Due to the exploratory nature of the study and the limited differences in mass loss observed among isolates, statistical analyses were primarily used to identify reproducible trends rather than to infer definitive degradation efficiencies. The collected mass-loss data were analyzed using IBM SPSS Statistics version 27.0 (IBM Corp., Armonk, NY, USA) with a one-way analysis of variance (ANOVA).

### Schiff’s reagent staining procedure for detecting oxidative surface modification

Schiff’s reagent staining was used as a preliminary qualitative indicator of oxidative surface modification in natural rubber discs. Sterilized natural rubber discs were stained with 10 mL of fuchsin reagent for 10–30 min at ambient temperature (20 °C). The excess reagent was removed, and 10 mL of sulfite solution was added to terminate non-specific reactions. A magenta coloration was interpreted as a possible indication of aldehyde-containing oxidation products on the rubber surface (Nayanashree and Thippeswamy 2015a, 2015b).

### Analysis of surface morphology using Scanning Electron Microscopy (SEM)

The surface morphology of incubated rubber discs was examined using a Phenom ProX G6 Desktop SEM. Samples were mounted on SEM stubs and observed at 5 kV to evaluate fungal colonization and surface-associated structural alterations (Xu et al. 2024).

### Morphological studies

Fungal colonies were cultured on PDA at room temperature (18–25 °C) for 4 weeks. Colony morphology (front and reverse) was documented. The front and reverse of the culture were photographed with an iPhone 14. Macro-morphological characteristics of fungal fruiting bodies were examined using a stereomicroscope (Leica S8AP0, Tokyo, Japan), while micro-morphological features were observed and photographed with a compound microscope (Olympus BX53, Tokyo, Japan). Measurements were made using Tarosoft(R) Image Frame Work software. Adobe Photoshop CC 2017 was used to prepare photo plates. Herbarium specimens were made from dry cultures. The culture plates were placed in a hot-air oven at 40 °C to dry. The cultures were continuously observed and removed once completely dry. Dried cultures were stored in envelopes for the preparation of herbarium specimens. Herbarium specimens were deposited at Guizhou Medical University (**GMB-W**) and Mae Fah Luang University Herbarium (**MFLU**). The living cultures were deposited in the Culture Collection of Guizhou Medical University (**GMBCC**), China, and the Mae Fah Luang University Culture Collection (**MFLUCC**), Thailand. MycoBank numbers (MB) were registered as outlined in MycoBank (2026). Data from taxa from the Greater Mekong Subregion are deposited in the database of Chaiwan et al. (2021).

## Phylogenetic analyses

### DNA extraction, PCR amplification, and sequencing

Genomic DNA was extracted directly from scraped fresh mycelia grown on 1-month-old artificial culture media (PDA), using an E.Z.N.A. Forensic DNA Kit (BIO-TEK), in accordance with the manufacturer’s protocol. The different gene regions, primers, and amplification protocols used are summarized in Table 2. Polymerase chain reaction (PCR) amplifications were performed using a 25 μL PCR mixture containing 8.5 μL ddH_2_O, 12.5 μL 2× Master Mix (Bioteke Corporation, Beijing, China), 2 μL DNA template, and 1 μL each of the forward and reverse primers (Tibpromma et al. 2018). Purification and sequencing of PCR products were performed at Bioteke, P.R. China.

**Table 2.** Loci, primers, and PCR amplification conditions used in this study.

Locus	Primers	PCR amplification conditions	References
ITS	ITS5/ITS4	Initial denaturation at 94 °C for 5 min; followed by 35 cycles of denaturation at 94 °C for 30 s, annealing at 51 °C for 1 min, and extension at 72 °C for 2 min; final extension at 72 °C for 10 min	White et al. 1990
LSU	LR0R/LR5		Vilgalys and Hester 1990
*rpb*2	fRPB2-5F/fRPB2-7cR	Initial denaturation at 94 °C for 5 min; followed by 35 cycles of denaturation at 94 °C for 30 s, annealing at 54 °C for 40 s, and extension at 72 °C for 1 min 20 s; final extension at 72 °C for 10 min	Liu et al. 1999
*tef*1-α	EF1/EF2	Initial denaturation at 94 °C for 5 min; followed by 35 cycles of denaturation at 94 °C for 45 s, annealing at 55 °C for 45 s, and extension at 72 °C for 1 min; final extension at 72 °C for 10 min	O’Donnell et al. 1998
*TUB*2	Bt2a/Bt2b	Initial denaturation at 94 °C for 2 min; followed by 35 cycles of denaturation at 94 °C for 30 s, annealing at 55 °C for 30 s, and extension at 72 °C for 1 min; final extension at 72 °C for 5 min	Glass and Donaldson 1995

### Phylogenetic tree construction

Sequences with high similarity (>90%) were identified by BLASTn searches to determine the closest matches to the taxa. The representative species used in the phylogenetic analysis were selected based on previous publications (Kaur et al. 2018; Sandoval-Denis et al. 2019; Liu et al. 2024, 2026) and GenBank (https://www.ncbi.nlm.nih.gov/). The accession numbers of the sequences used in this study are shown in Suppl. material 1: tables S2–S4. Initial sequence alignments were processed with MAFFT v.7 (http://mafft.cbrc.jp/alignment/server) using default settings (Katoh et al. 2019). The sequences were trimmed using TrimAl v. 1.2 with the “gappyout” automated trimming option (Capella-Gutiérrez et al. 2009). The alignments were visually checked and manually improved where necessary. The multiple-locus matrix was concatenated using Sequence Matrix (Vaidya et al. 2011). Multilocus phylogenetic analyses of the concatenated genes were conducted using maximum likelihood (ML) and Bayesian inference (BI). The CIPRES Science Gateway portal (Miller et al. 2012) was used to run both RAxML and Bayesian analyses. ML analysis was performed with RAxML-HPC2 on the XSEDE v.8.2.10 tool (Stamatakis 2014), using the GTR+GAMMA substitution model with 1,000 bootstrap replicates. Bayesian analysis was performed with MrBayes v.3.0b4 (Huelsenbeck and Ronquist 2001), using the best-fit model of sequence evolution estimated with MrModelTest 2.2 (Nylander 2004). MrBayes analyses were performed with GTR+I+G for 1 million generations, sampling every 100^th^ generation and ending the run automatically when the standard deviation of split frequencies dropped below 0.01, with a 25% burn-in. Phylograms were visualized using FigTree v.1.4.0 (Rambaut 2012) and edited in Microsoft PowerPoint 2021 and Adobe Illustrator CC 2019.

## Results

### Isolation and preliminary screening of enzyme-producing fungi

**Figure 1.**
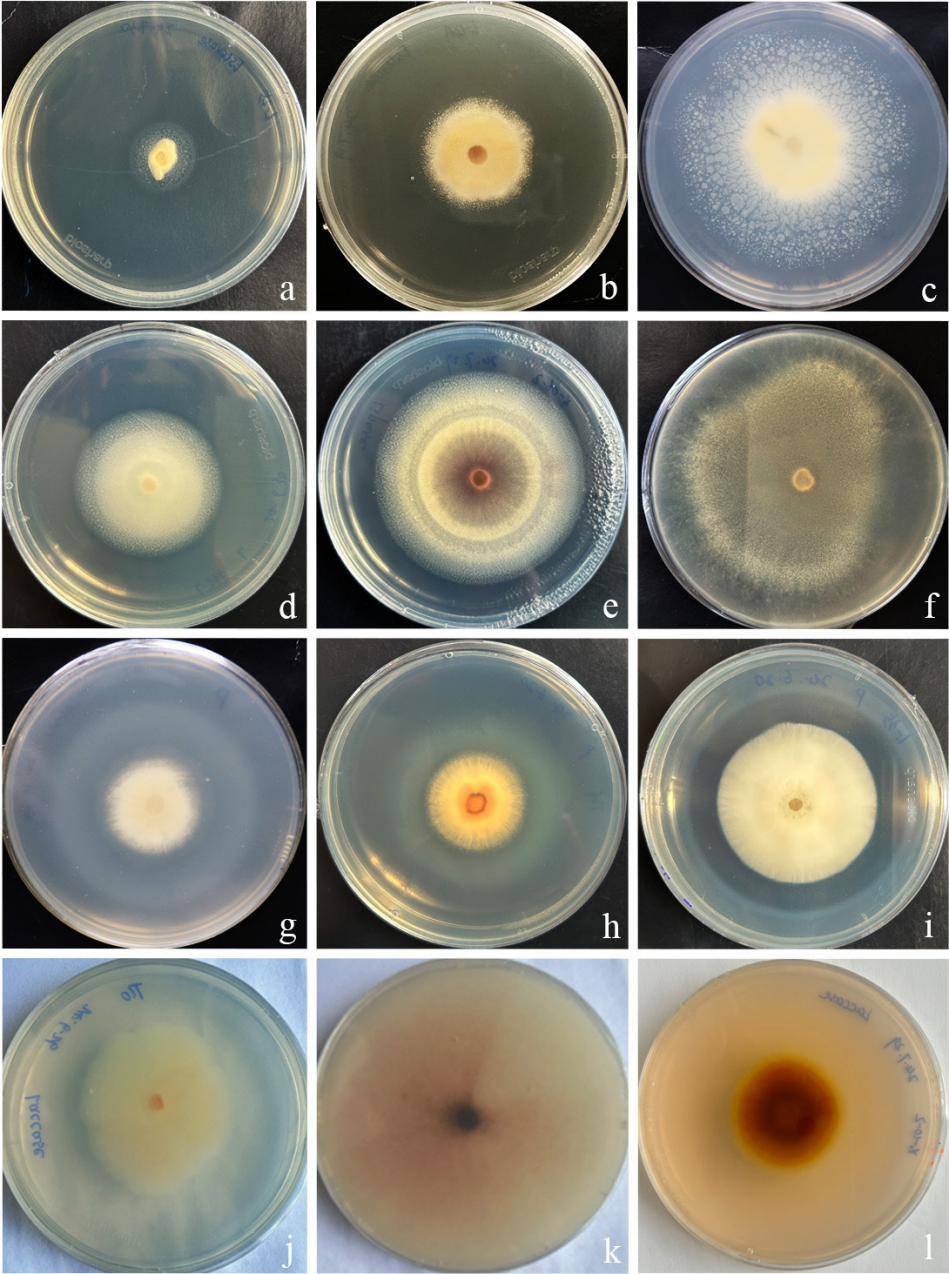
Representative images of positive enzyme activities. **a–c**. Esterase; **d–f**. Lipase; **g–i**. Protease; **j–l**. Laccase.

Sixty-nine fungal isolates were obtained from various samples. Preliminary qualitative enzyme screening identified 36 enzyme-producing isolates. Esterase activity was observed in 35 isolates, lipase activity in 20 isolates, protease activity in four isolates, and laccase activity in three isolates. Several isolates showed positive reactions for multiple enzyme activities. No isolate produced all four enzymes simultaneously. Four isolates, 5-1, X-10-2, T10, and L-12A, showed positive reactions for three enzyme activities: 5-1 and L-12A showed esterase, lipase, and protease activities, while X-10-2 and T10 showed esterase, lipase, and laccase activities. In addition, isolates L-35 and T21 showed both esterase and protease activities, while 15 isolates showed both esterase and lipase activities. Thirteen isolates showed only esterase activity, and one isolate showed only lipase activity. Representative enzyme reactions are shown in Fig. 1, and the distribution of enzymatic activities among the isolates is summarized in Table 3.

**Table 3.** Isolates of enzyme-producing fungi and associated enzymatic activities; N/A indicates that no enzyme production was detected.

Isolate strain no.	Esterase	Lipase	Protease	Laccase
1-1C	1.52	0.24	N/A	N/A
5-1	1.34	1.08	1.52	N/A
G4	1.61	1.18	N/A	N/A
L-11A	1.24	N/A	N/A	N/A
L-12A	1.22	0.72	1.42	N/A
L-12B	0.80	0.71	N/A	N/A
L-13	1.25	N/A	N/A	N/A
L-17	0.61	0.35	N/A	N/A
L-23B	1.50	1.04	N/A	N/A
L-24	0.79	0.57	N/A	N/A
L-25	0.50	0.77	N/A	N/A
L-28	1.23	N/A	N/A	N/A
L-33	0.79	0.44	N/A	N/A
L-34	0.82	N/A	N/A	N/A
L-35	0.68	N/A	1.47	N/A
L-36	1.50	0.85	N/A	N/A
L-37B	1.38	N/A	N/A	N/A
L-38	1.76	1.85	N/A	N/A
X-01	0.80	1.22	N/A	N/A
X-07	1.11	1.14	N/A	N/A
X-10-2	1.61	1.21	N/A	Reddish brown
T2	0.82	0.85	N/A	NA
T5	0.61	N/A	N/A	Reddish brown
T6	0.88	N/A	N/A	N/A
T8	0.28	N/A	N/A	N/A
T9	N/A	1.75	N/A	N/A
T10	1.4	1.08	N/A	Reddish brown
T15	1.64	1.30	N/A	N/A
T16-2	1.29	N/A	N/A	N/A
T21	1.21	N/A	1.69	N/A
T24	0.65	N/A	N/A	N/A
T25	0.31	0.12	N/A	N/A
T26	0.50	N/A	N/A	N/A
T29	1.00	N/A	N/A	N/A
T32	0.19	N/A	N/A	N/A
T36	0.15	N/A	N/A	N/A

**Figure 2.**
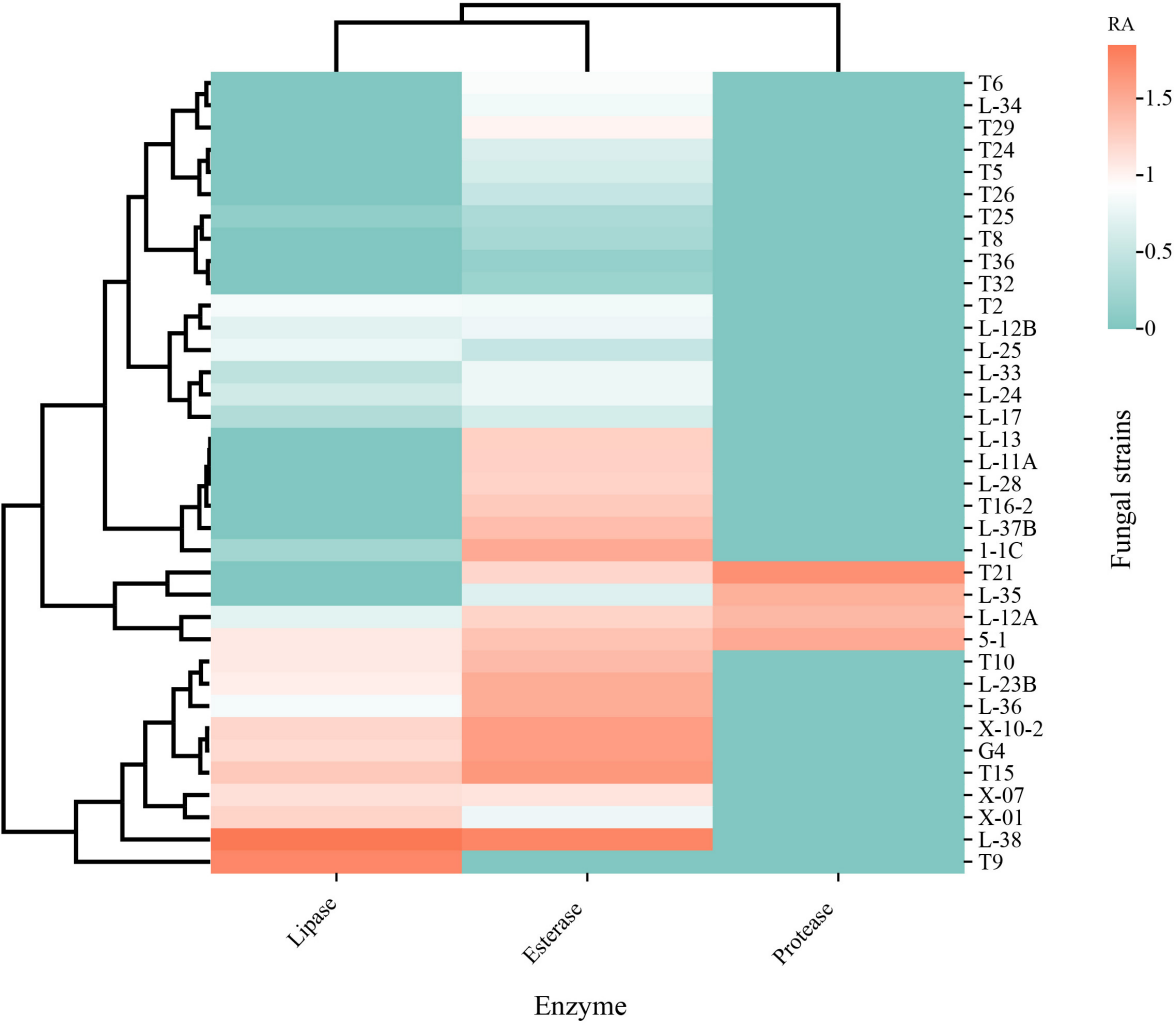
Clustered heatmap of enzyme activity values obtained for lipase, esterase, and protease enzymes from different strains.

Based on the qualitative screening results presented in Table 3, the clustered heatmap generated in Origin shows the activity distribution of 36 enzyme-producing strains, namely esterase, lipase, and protease activities. The vertical axis in the figure represents strains and their clustering groups, and the horizontal axis corresponds to the three enzyme activities (Fig. 2). The color gradient from orange-red to green indicates the relative extent of detectable enzyme reactions, with orange-red representing comparatively stronger reactions and green representing weaker or absent reactions. Laccase activity was excluded from the heatmap because it was evaluated only by qualitative reddish-brown color formation rather than by measurable halo-zone comparison. This overview summarizes the enzyme activity results across all tested strains. In the esterase screening assay, strains 1-1C, G4, L-12A, L-12B, L-13, L-23B, L-24, L-25, L-28, L-36, L-37B, L-38, X-07, X-10-2, T10, T15, T16-2, and T-21 showed enzyme zones larger than the colony zones, while the other 17 strains showed enzyme zones smaller than the colony zones, and one strain showed no enzyme production. For the lipase test, strains 5-1, G4, L-11A, L-12B, L-23B, L-38, X-07, X-01, X-10-2, T9, T10, and T15 showed enzyme zones larger than the colony zones, while the other eight strains showed enzyme zones smaller than the colony zones, and 16 strains showed no enzyme production. Protease production was detected only in four strains: 5-1, L-12A, L-35, and T-21, with the remaining 32 strains being inactive. Similarly, laccase activity, indicated by reddish-brown color, was positive in three strains, T5, T10, and X-10-2, while the other 33 strains showed no production. As these enzymatic assays were conducted as preliminary qualitative screening tests, the results should be interpreted as indicative of potential extracellular enzyme production rather than quantitative measurements of enzymatic activity.

### Mass-loss assessment of natural rubber by selected fungal isolates

Thirty-six enzyme-producing isolates were evaluated for their capacity to modify natural rubber discs over a 2-month incubation period. Most isolates exhibited minimal or no detectable weight reduction (Suppl. material 1: table SS1). Four isolates (L-12A, L-25, L-33, and T-21) consistently demonstrated measurable mass loss across all three replicates and were selected for further analysis. The observed weight loss ranged from 0.77% ± 0.27% to 1.24% ± 0.25% (Table 4). Isolate T-21 exhibited the highest average mass loss (1.24% ± 0.25%), followed by L-12A (1.03% ± 0.14%), L-33 (0.82% ± 0.29%), and L-25 (0.77% ± 0.27%) (Table 4).

**Table 4.** Weight loss of natural rubber discs after inoculation with different fungal strains.

**Isolate strains**	**Initial weight (M1, g)**	**Final weight (M2, g)**	**Difference (g)**	**Weight loss percentage (M, 100%)**	**Average**	**Standard deviation**
L-12A	4.23	4.19	0.04	0.95%	1.03%	0.14%
	4.21	4.17	0.04	0.95%		
	4.19	4.14	0.05	1.19%		
L-25	4.35	4.31	0.04	0.92%	0.77%	0.27%
	4.34	4.32	0.02	0.46%		
	4.33	4.29	0.04	0.92%		
L-33	5.32	5.26	0.06	1.13%	0.82%	0.29%
	5.32	5.28	0.04	0.75%		
	5.29	5.26	0.03	0.57%		
T21	4.60	4.55	0.05	1.09%	1.24%	0.25%
	4.57	4.50	0.07	1.53%		
	4.55	4.50	0.05	1.10%		

**Figure 3.**
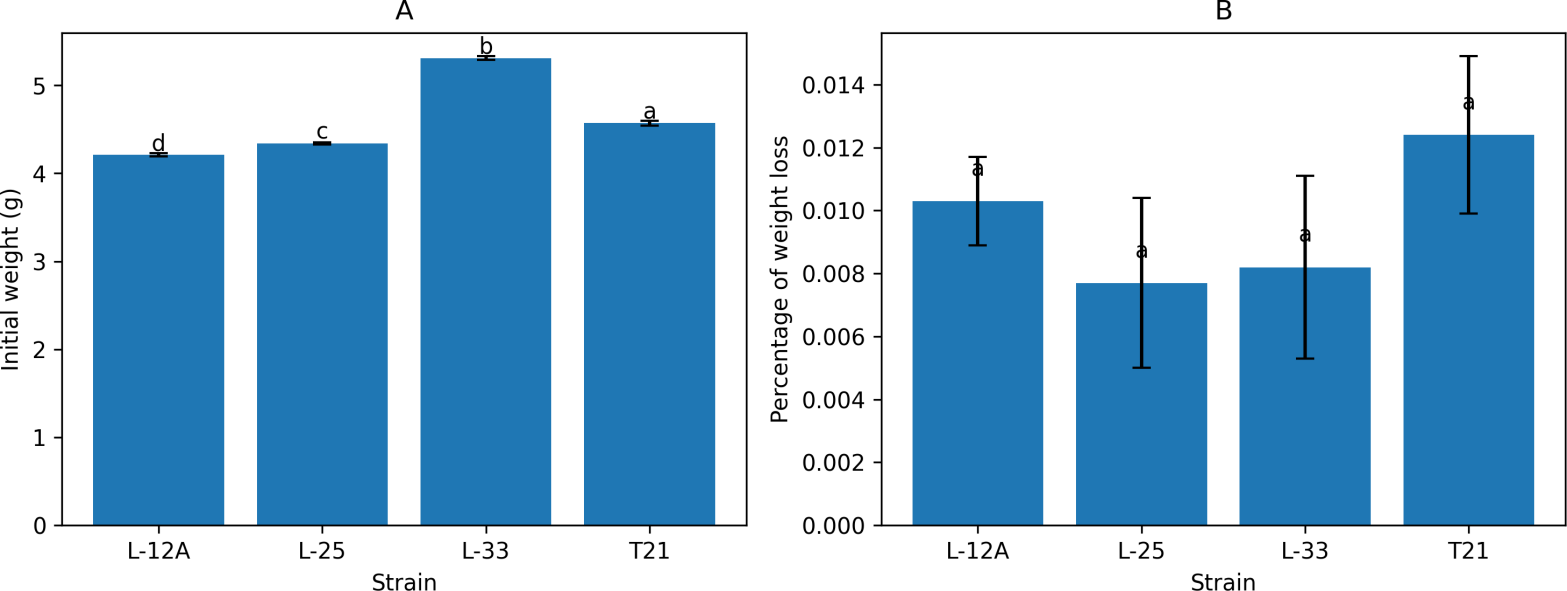
One-way ANOVA of weight-loss measurements of natural rubber discs incubated with different fungal strains. **A**. Initial weights of rubber discs showing significant differences among groups; different letters indicate *p* < 0.05; **B**. Weight-loss percentages after incubation. No statistically significant differences were detected among strains; the same letters indicate *p* > 0.05. Error bars represent standard deviation (*n* = 3).

In addition, four strains were tested using one-way ANOVA, which revealed significant differences in the initial weight of rubber discs among strain groups (*F* (3, 8) = 4.21, *p* = 0.042; *p* < 0.05). However, no significant effect of initial weight differences was observed on the final mass loss (*F* (3, 8) = 2.662, *p* = 0.119; *p* > 0.05). The tested strains showed overlapping standard deviations and confidence intervals for mass-loss measurements, and no statistically significant differences were detected among treatments (Fig. 3). These observations indicate relatively similar measurable trends among strains under the experimental conditions rather than clear differences in rubber deterioration activity. These findings suggest that the tested fungi induced limited mass loss during incubation, which is consistent with early-stage surface modification rather than extensive degradation of the rubber matrix.

### Detection of oxidative surface modification using Schiff’s reagent

**Figure 4.**
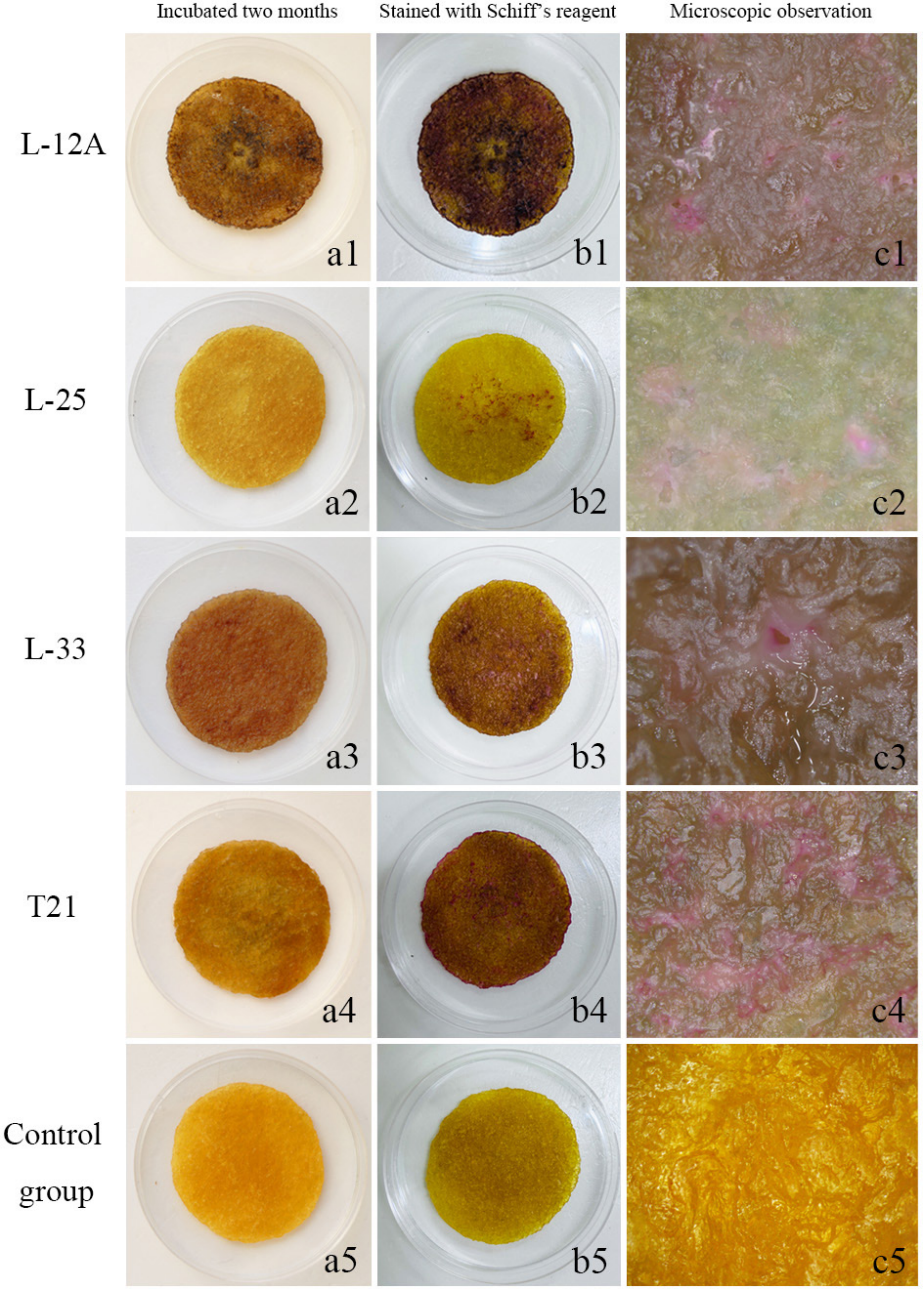
Schiff’s reagent staining of natural rubber showing oxidative surface changes. **a1–a5**. After 2 months of incubation, the rubber discs were washed and dried; **b1–b5**. Rubber discs stained with Schiff’s reagent; **c1–c5**. The stained area observed under magnification.

Natural rubber discs inoculated with the four positive fungal strains developed a distinct purple color upon staining with Schiff’s reagent. The staining response may indicate the presence of aldehyde-containing oxidation products resulting from surface-level oxidative modification of the rubber material. No color change was observed in the control discs (Fig. 4).

### SEM observation of surface alteration of natural rubber

**Figure 5.**
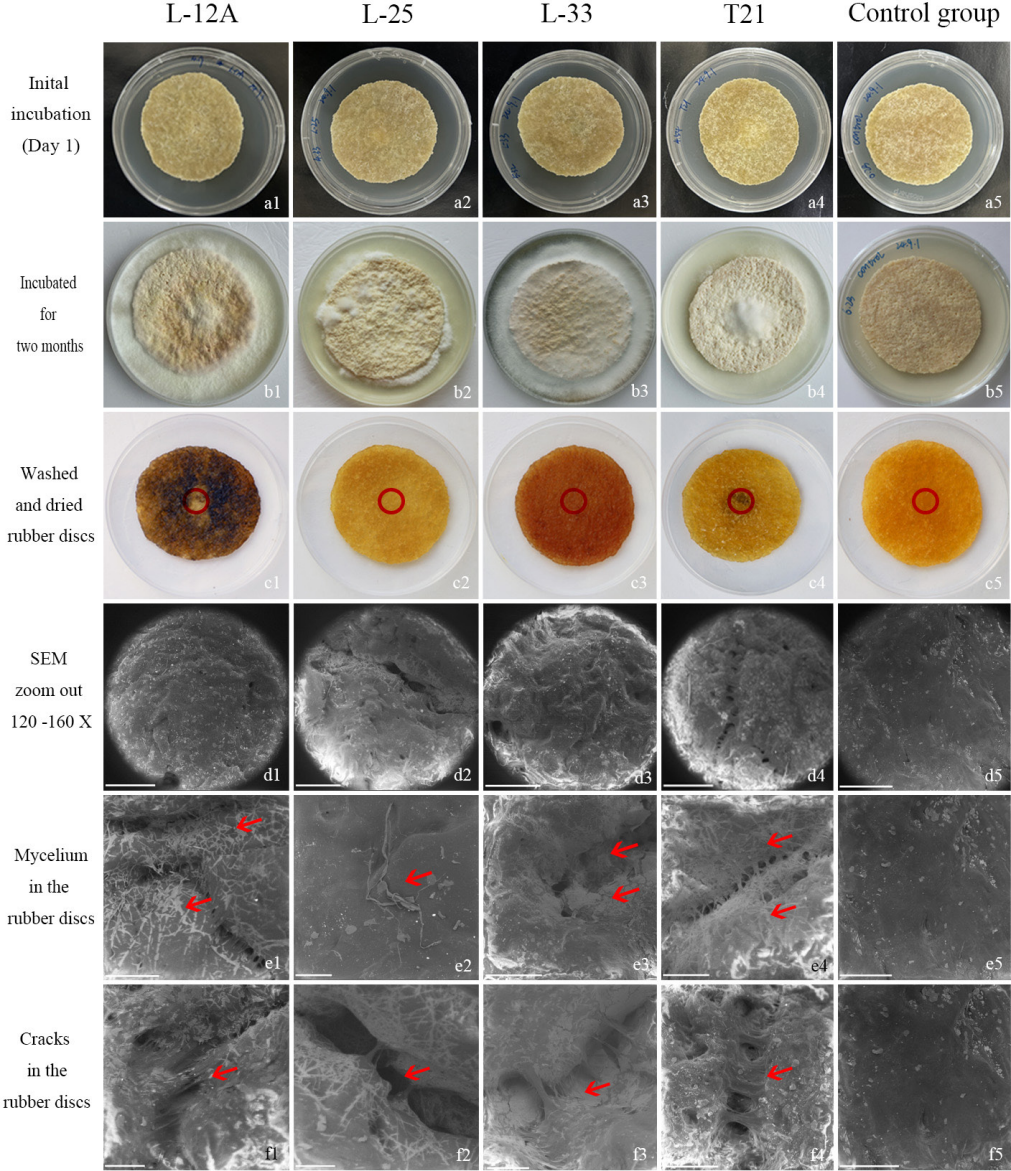
SEM observations of surface alteration and fungal colonization on natural rubber discs after fungal incubation. **a1–a5**. Rubber discs after 1 day of incubation; **b1–b5**. Rubber discs after 2 months of fungal incubation; **c1–c5**. Rubber discs after washing and drying prior to SEM analysis (red circles indicate sampled regions); **d1–d5**. SEM images of rubber disc surfaces; **e1–e4**. Fungal hyphae and mycelial networks observed on rubber surfaces (red arrows indicate fungal structures); **f1–f4**. Surface fissures and cracks observed on rubber discs after fungal incubation (red arrows indicate cracks); **d5, e5, f5**. Uninoculated control discs observed under SEM. Scale bars: 500 μm (**d1–d5**), 200 μm (**e1**, **f4**), 300 μm (**e3**, **e5**, **f5**), 100 μm (**e2**, **e4**, **f1–f3**).

Fungal colonization and hyphal growth were evident on the surfaces of natural rubber discs during incubation. Surface alterations, such as discoloration and splitting, predominantly occurred in the inoculated treatments, while the uninoculated control discs exhibited no significant visible changes. SEM analysis revealed fungal proliferation on the rubber surfaces, accompanied by irregular fissures, erosion-like features, and crack formation in the inoculated samples (Fig. 5). In contrast, the control discs retained a relatively smooth surface structure without visible cracking or fungal colonization. These observations indicate that fungal incubation was associated with detectable surface alterations and morphological changes in the natural rubber material under the experimental conditions.

### Phylogenetic analyses

**Figure 6.**
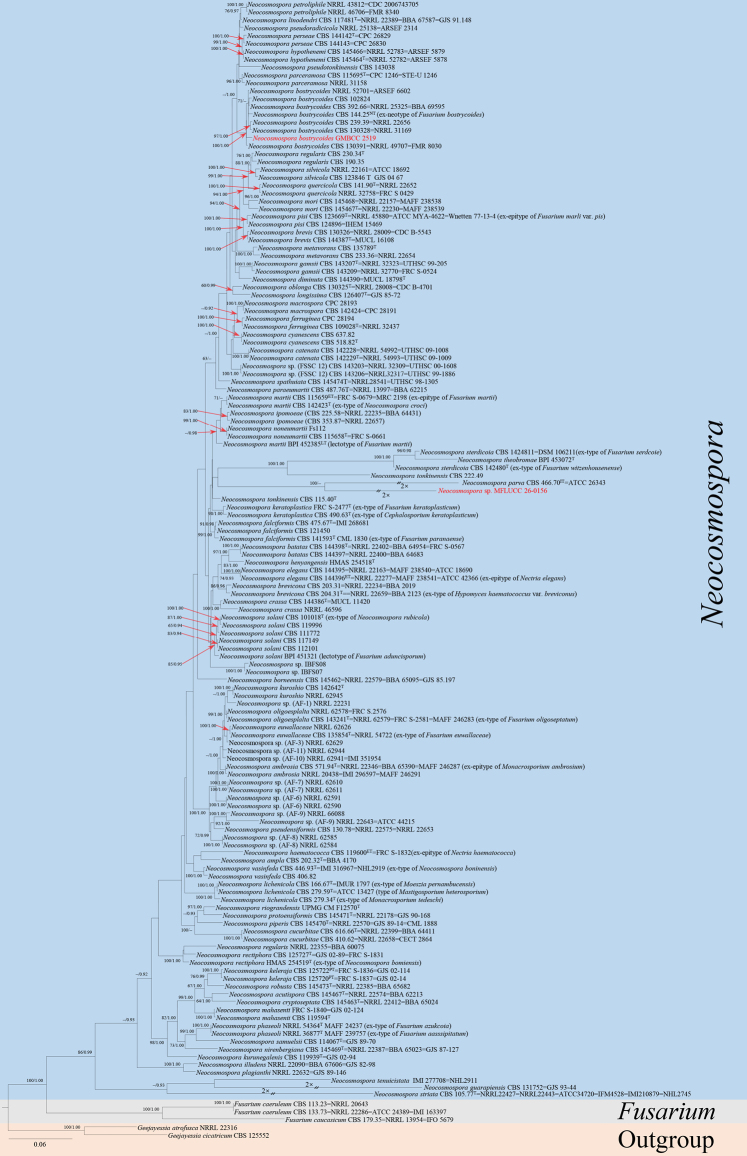
Phylogram generated from maximum likelihood analysis based on combined ITS, LSU, *rpb*2, and *tef*1-α sequences of 145 strains. Bootstrap values (ML ≥ 60%) and Bayesian posterior probabilities (BYPP ≥ 0.90) are shown at the nodes. New isolates are indicated in red; ex-type strains are marked with “T.”

Analysis 1: A multilocus phylogenetic analysis of the genus *Neocosmospora* was conducted using ITS, LSU, *rpb*2, and *tef*1-α gene sequences, encompassing 145 strains. Phylogenetic analysis placed the strain GMBCC2519 within the *Neocosmospora bostrycoides* clade with 100% ML and 1.00 BYPP statistical support, confirming its identification as *N. bostrycoides*. Another strain, MFLUCC 26–0156, clustered with *N. parva* (CBS 466.70) with 100% ML support but had BYPP support below 0.90 and a long branch (Fig. 6).

**Figure 7.**
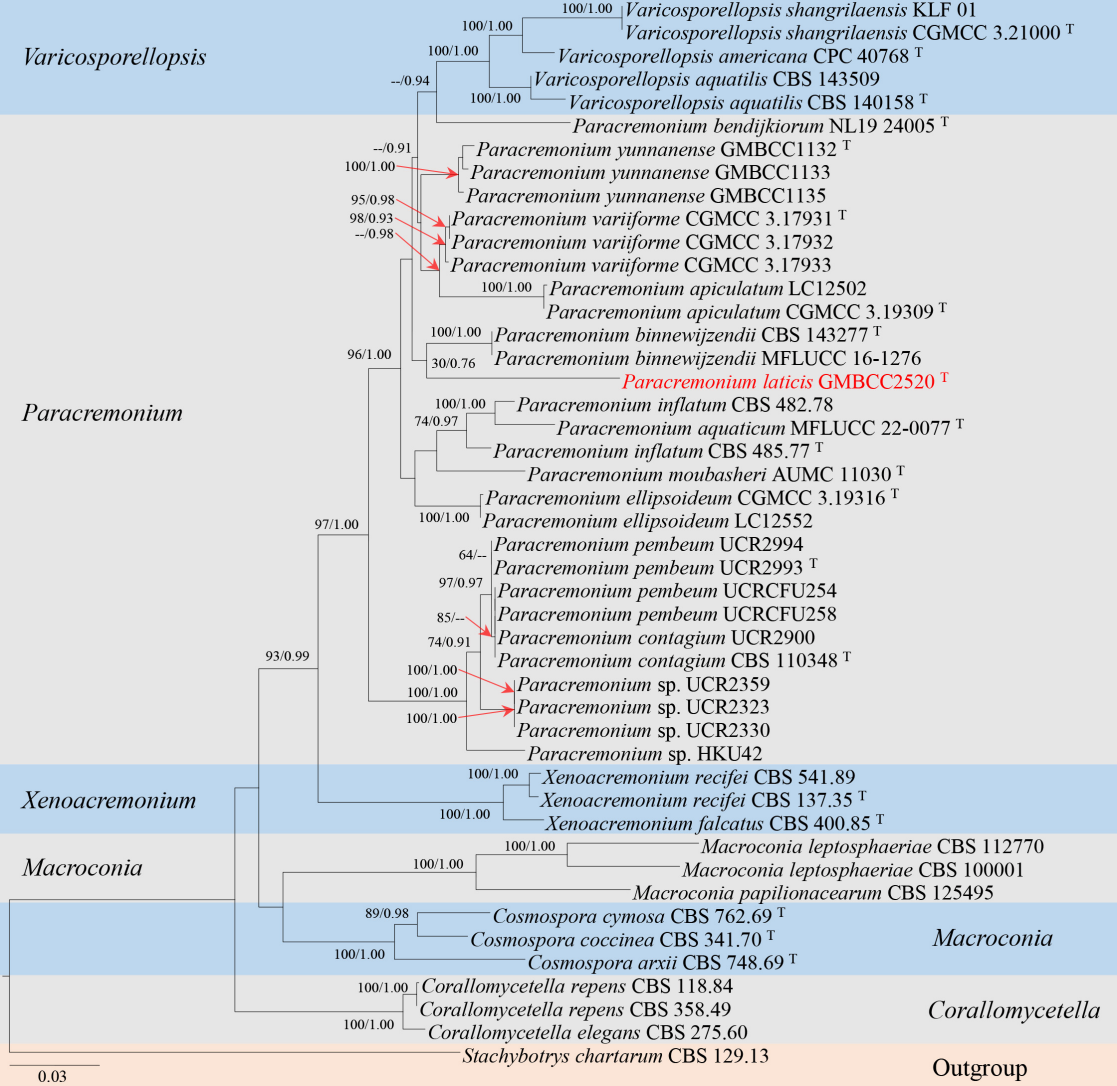
Phylogram generated from maximum likelihood analysis based on combined ITS, LSU, and *TUB2* sequences of 46 strains. Maximum likelihood bootstrap values (ML) and Bayesian posterior probabilities (BYPP) are indicated at the nodes. New isolates are indicated in red; ex-type strains are marked with “T.”

Analysis 2: Phylogenetic analysis of *Paracremonium* was performed using combined ITS, LSU, and *TUB2* sequence data from 46 strains. The resulting phylogeny showed that strain GMBCC2520 formed an independent lineage closely related to *P. binnewijzendii* strains, although the statistical support was low (ML 30% and BYPP 0.76) (Fig. 7).

**Figure 8.**
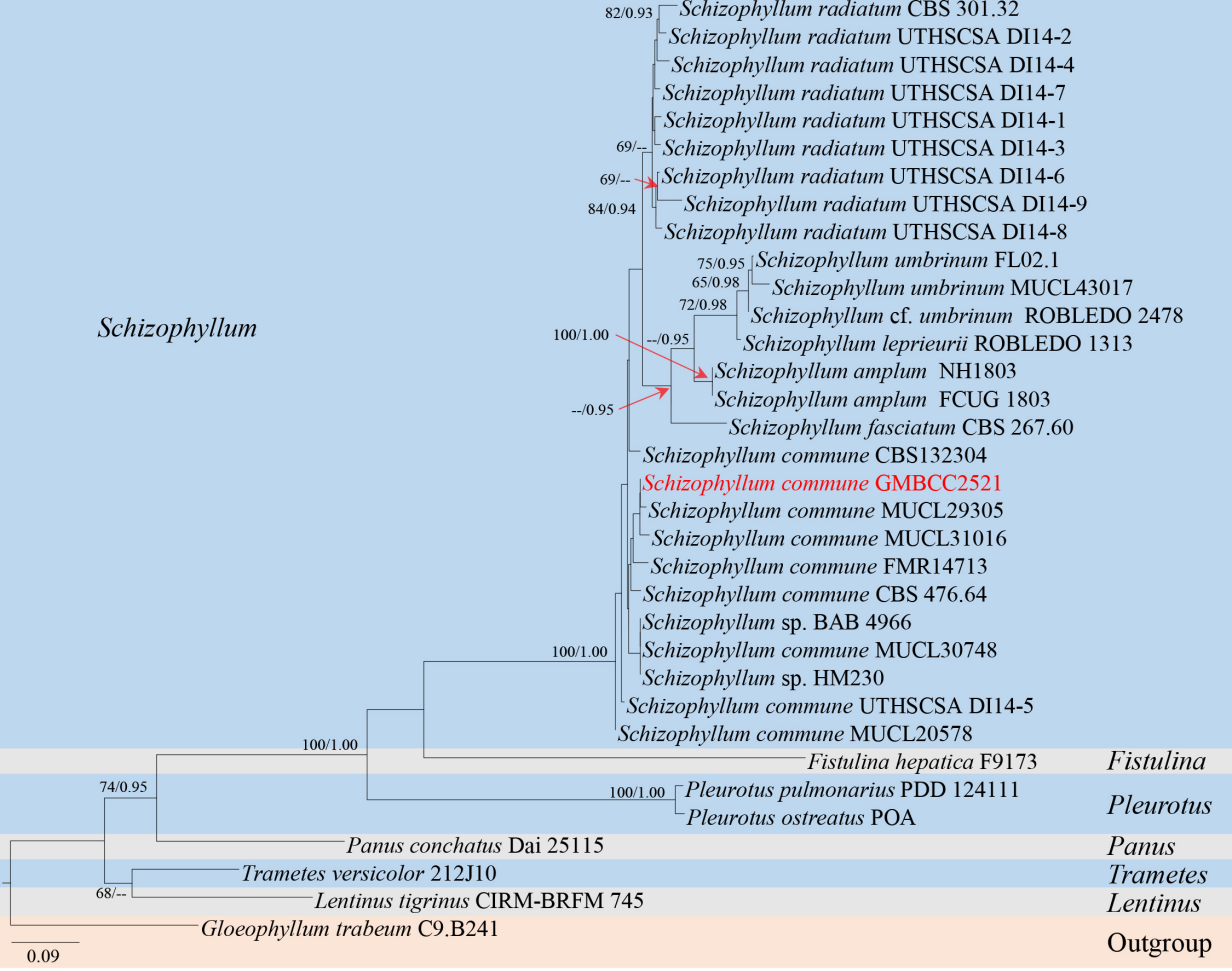
Phylogram generated from maximum likelihood analysis based on combined ITS, LSU, *tef*1-α, and *rpb*2 sequences of 34 strains. Bootstrap values (ML ≥ 60%) and Bayesian posterior probabilities (BYPP ≥ 0.90) are shown at the nodes. New isolates are indicated in red.

Analysis 3: Combined ITS, LSU, *tef*1-α, and *rpb*2 sequence analyses of 34 strains identified strain GMBCC2521 as *Schizophyllum commune* (Fig. 8). The ML and Bayesian analyses generated similar topologies.

## Taxonomy

### *Neocosmospora* E.F. Sm., Bull. U.S.D.A. 17: 45. 1899

#### Neocosmospora bostrycoides

Taxon classificationFungiSordariomycetesNectriaceae

(Wollenw. & Reinking) Sandoval-Denis, L. Lombard and Crous, Persoonia 43: 115 (2019)

AF523CB9-E416-5B44-BBBB-73585BB527A4

831174

Fig. 9

##### Description.

Associated with contaminated rubber latex. **Sexual morph**: Undetermined. **Asexual morph on PDA: *Conidiophores*** abundant on aerial and substrate mycelium, erect and simple, straight or flexuous, smooth- and thin-walled, bearing terminal or lateral, forming monophialides on the apices. ***Phialides*** (11–) 15 × 30 (–35) × 2.5–5 μm (x– = 22 × 4 μm, *n* = 30), subcylindrical to subulate, monophialidic, smooth- and thin-walled with a short flared apical collarette. ***Aerial conidia*** 5–11 × 2.5–5 μm (x– = 6.8 × 3.6 μm, *n* = 50), obovoidal, broadly ellipsoidal to ellipsoidal, straight or rarely curved, 0(–1)-septate, hyaline, smooth- and thin-walled, clustering abundantly in false heads at the tip of monophialides. ***Sporodochial conidiophores*** sparingly verticillately branched, bearing terminal whorls of 2–3-monophialides. ***Sporodochial phialides*** 11–20 × 3–4 μm (x– = 15 × 3.4 μm, *n* = 30), flask-shaped, subcylindrical to short subulate, smooth- and thin-walled, conidiogenous loci with inconspicuous periclinal thickening and collarette. ***Sporodochial conidia*** 22–38 × 4–6 μm (x– = 31 × 4.6 μm, *n* = 50), moderately to distinctly dorsiventrally curved narrowing gently toward base, dorsal line usually almost straight; apical cell often equal in length to adjacent cell, blunt and rounded with curved apex; basal cell often distinctly notched, 1–4-septate, hyaline, smooth- and thick-walled. ***Chlamydospores*** 6–10 μm diam., abundantly formed, globose to subglobose, smooth- and thick-walled, terminal or intercalary in hyphae or conidia, solitary, in chains or in clusters.

**Figure 9.**
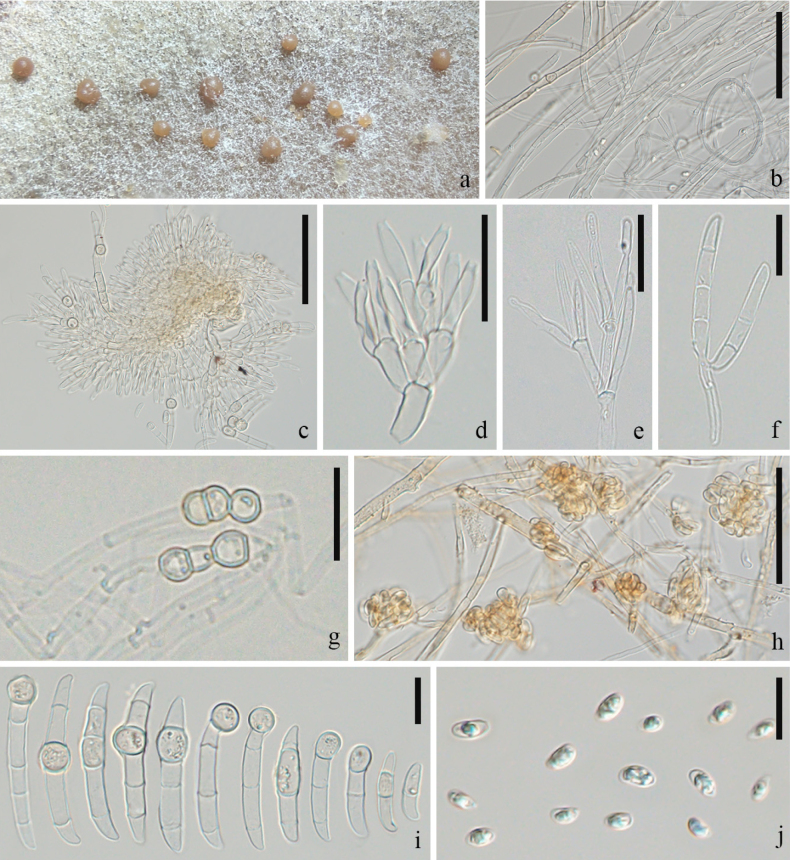
*Neocosmospora bostrycoides* (GMB-W1263). **a**. Sporodochia formed on the surface of PDA after 8 weeks; **b**. Mycelium; **c–f**. Sporodochial conidiophores; **g**. Chlamydospores; **h**. Aerial conidiophore with conidia; **i**. Sporodochial conidia with chlamydospores; **j**. Aerial microconidia. Scale bars: 50 μm (**b**, **c**, **h**), 20 μm (**d**, **e**, **g**), 10 μm (**f**, **i**, **j**).

##### Culture characteristics.

Colonies on PDA at room temperature (20–25 °C) covered the entire 6 cm Petri dish within three days, white to pale yellow, with abundant aerial mycelium; reverse white to pale yellow. Sporulation was observed after prolonged incubation on PDA at room temperature.

##### Material examined.

China, • Yunnan Province, Lincang City, contaminated rubber latex, 28 July 2022, Rui-Fang Xu, L-12A (GMB-W1263), living culture GMBCC2519.

##### Notes.

Phylogenetic analysis placed strain GMBCC2519 within the *Neocosmospora bostrycoides* lineage, supported by 100% ML and 1.00 BYPP values (Fig. 6). Morphologically, GMB-W1263 closely resembles the reference strain *Neocosmospora bostrycoides* (CBS 130391). It aligns with the species description but is characterized by shorter sporodochial conidia (22–38 μm vs. 40.5–51.5 μm) (Sandoval-Denis et al. 2019). Nucleotide comparisons revealed differences of 5 bp in ITS (2 gaps), 3 bp in LSU (0 gaps), and 6 bp in *rpb*2 (0 gaps) between GMBCC2519 and *Neocosmospora bostrycoides* (CBS 144.25). *Neocosmospora bostrycoides* was collected from contaminated old rubber latex in a container near a rubber tree. In the present study, this species showed measurable surface-associated changes on natural rubber under laboratory incubation conditions.

#### Neocosmospora

Taxon classificationFungiSordariomycetesNectriaceae

sp.

39BDF801-C3D4-5C0D-8ED4-ED23625CFD67

Fig. 10

##### Description.

Associated with contaminated rubber latex. **Sexual morph**: Undetermined. **Asexual morph**: *Fruiting bodies* observed on the PDA plate covered with a natural rubber disc. ***Mycelium*** 3–7 μm wide, hyaline, septate, granulate. ***Conidiophores*** borne on aerial mycelium, slightly tapering upward, micronematous, mononematous, erect, simple, straight or slightly flexuous, smooth-walled, thin-walled, hyaline, sometimes reduced to conidiogenous cells. ***Conidiogenous cells*** 23–100 (–108) × 3–9 μm (x– = 65 × 4, *n* = 25), solitary or sympodial, integrated, determinate, terminal, hyaline, on substrate mycelium. ***Conidia*** 9–27 × 3–6 μm (x– = 15 × 4.5, *n* = 70), solitary, hyaline, ellipsoidal, acrogenous, simple, smooth-walled, 0–1-septate, straight to curved, reniform. ***Chlamydospores*** 7–13 μm diam., abundantly formed, globose to subglobose, smooth- and thick-walled, terminal or intercalary in hyphae, in chains or in clusters.

**Figure 10.**
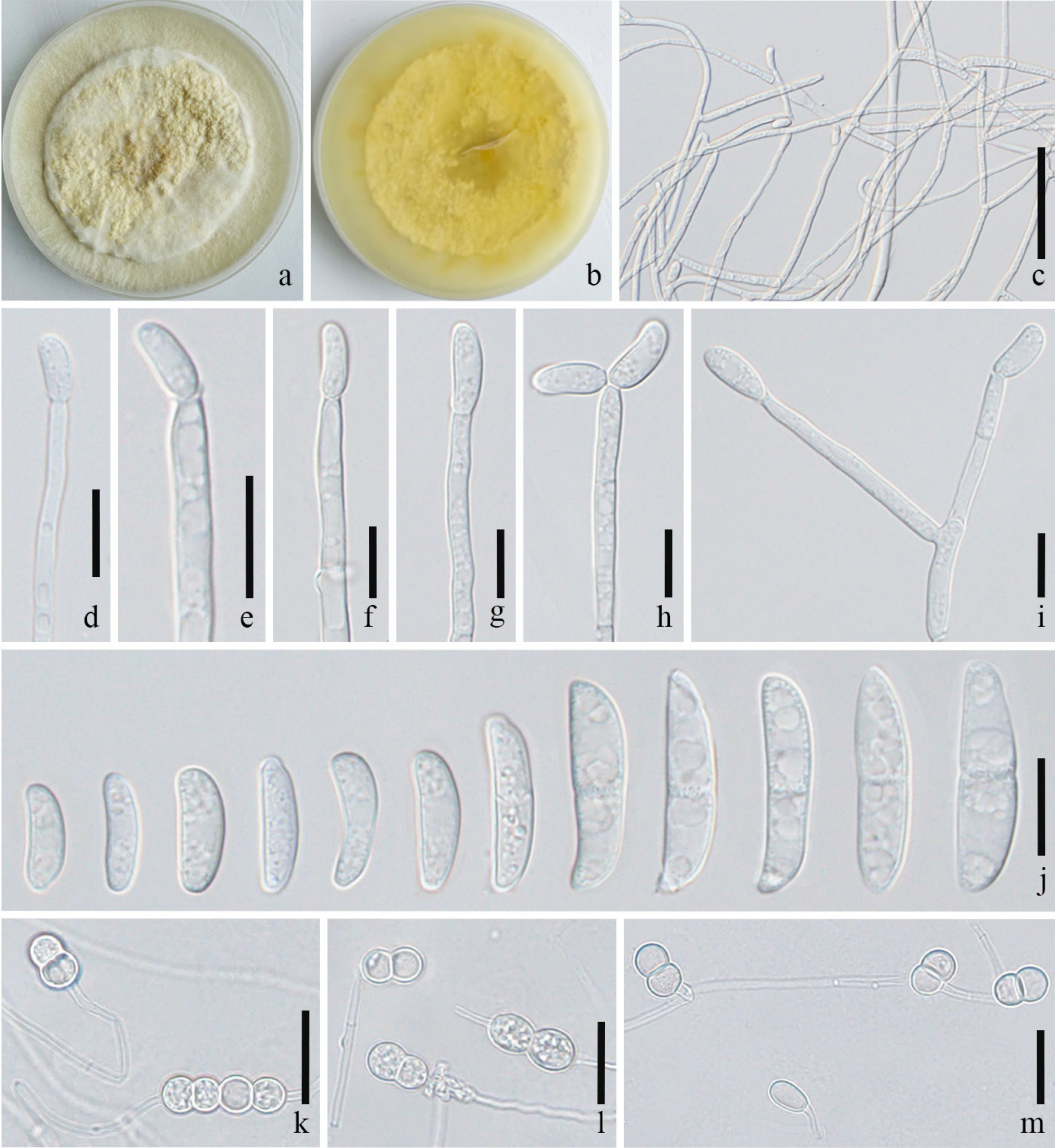
*Neocosmospora* sp. (MFLU 26–0038). **a, b**. Colonies observed on the PDA plate equipped with a natural rubber disc; **c**. Mycelium; **d–i**. Conidiogenous cells with conidia; **j**. Conidia; **k–m**. Chlamydospores. Scale bars: 50 μm (**c**), 10 μm (**d–j**), 10 μm (**k–m**).

##### Culture characteristics.

Colonies on PDA at room temperature (20–25 °C) covered the entire plate within one week, white to pale yellow with aerial mycelium. Sporulation was observed after incubation on PDA covered with natural rubber discs.

##### Material examined.

Thailand, • Chiang Rai Province, contaminated rubber latex, 19 December 2023, Rui-Fang Xu, T21 (MFLU 26–0038), living culture MFLUCC 26–0156.

##### Notes.

Phylogenetically, strain MFLUCC 26–0156 clustered near *Neocosmospora parva* with 100% ML and low BI support (Fig. 6). Although ITS and LSU sequences showed high similarity to species within the *Neocosmospora solani* species complex, the absence of *tef*1-α sequence data prevented reliable species delimitation. The *tef*1-α gene is crucial for the molecular identification and phylogenetic analysis of *Neocosmospora* species, especially when combined with ITS and *rpb*2 genes (Zhang et al. 2024; Ismail et al. 2025). These genes together offer high taxonomic resolution, helping to distinguish closely related species within the genus. Therefore, this isolate is classified as *Neocosmospora* sp. until sufficient evidence is available to determine its species. This isolate was collected from a container located near a rubber tree during the productive phase in Chiang Rai, Thailand. This is the first observation that *Neocosmospora* sp. can cause oxidative surface alteration of natural rubber.

### *Paracremonium* L. Lombard & Crous.

#### Paracremonium laticis

Taxon classificationFungiSordariomycetesNectriaceae

R.F. Xu, K.D. Hyde & Tibpromma sp. nov.

2BE01837-0F61-5951-B3A2-E99DFC3B7E27

905338

Fig. 11

##### Etymology.

Refers to the isolated source “rubber latex.”

**Figure 11.**
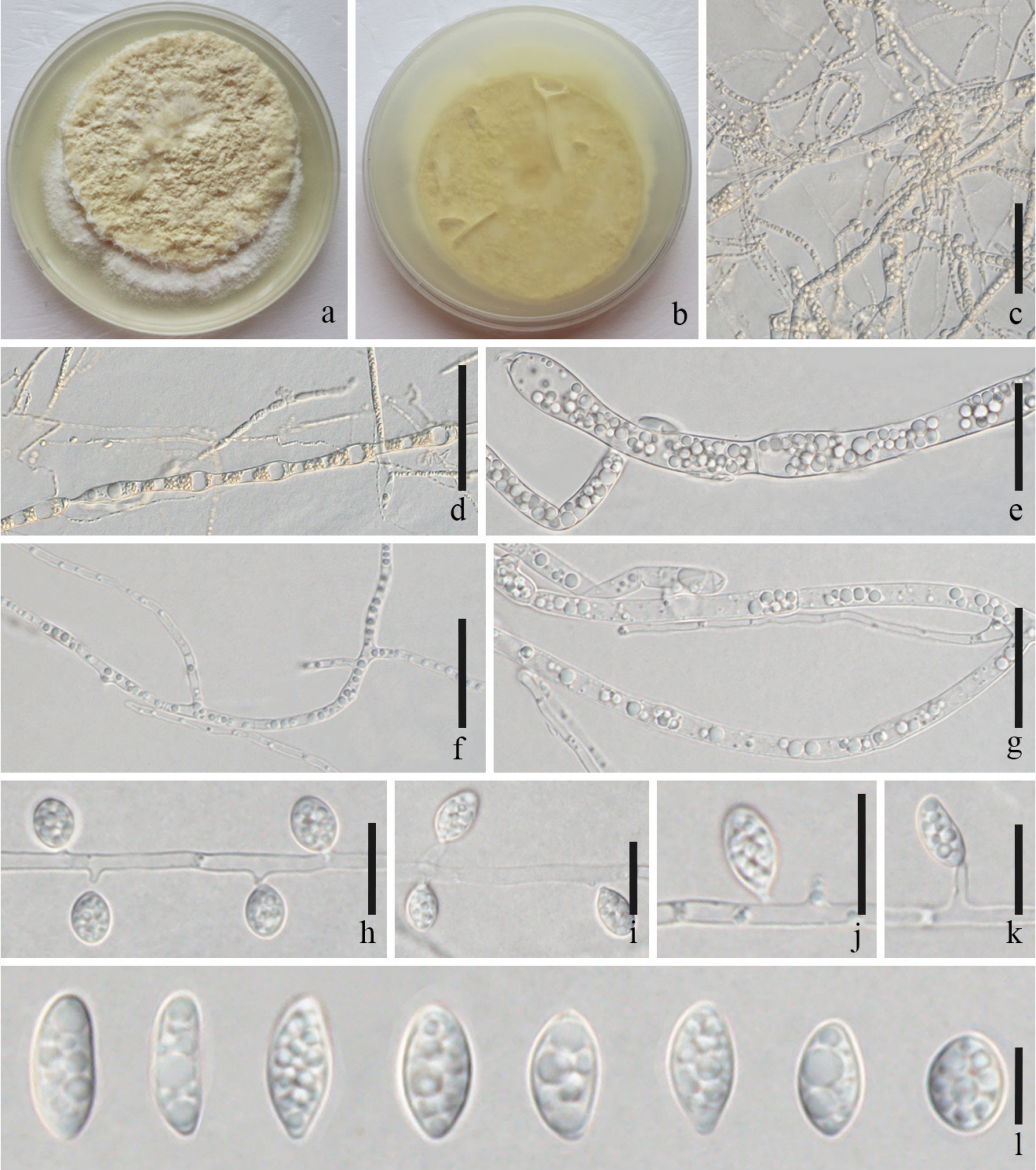
*Paracremonium laticis* (GMB-W1264). **a, b**. Colonies observed on the PDA plate equipped with a natural rubber disc; **c**. Mycelium; **d–g**. Hyphae; **h–k**. Conidiogenous cells with conidia; **l**. Conidia. Scale bars: 50 μm (**c, d**), 30 μm (**e**), 20 μm (**f, g**), 10 μm (**h–k**), 5 μm (**l**).

##### Holotype.

GMB-W1264.

##### Description.

Associated with contaminated rubber latex. **Sexual morph**: Undetermined. **Asexual morph: *Fruiting bodies*** observed on the PDA plate covered with a natural rubber disc. ***Mycelium*** 3–7 μm wide, hyaline, septate, granulate. ***Conidiophores*** borne on aerial mycelium, slightly tapering upward, micronematous, mononematous, erect, simple, straight or slightly flexuous, smooth-walled, thin-walled, hyaline, sometimes reduced to conidiogenous cells. ***Conidiogenous cells*** formed singly or integrated within hyphae, mostly enteroblastic, phialidic, arising laterally or terminally from hyphae, hyaline, smooth-walled, cylindrical to subcylindrical, occasionally slightly swollen at the base, tapering towards the apex, producing conidia successively. ***Conidiogenous loci*** are inconspicuous, without conspicuous collarettes. ***Conidia*** 5–10 × 3–6 μm (x– = 7.2 × 4.5, *n* = 50), solitary, hyaline, ellipsoidal to ovoid, sometimes slightly oblong, straight, smooth-walled, aseptate, typically rounded at both ends, slightly broader at the center with granular or guttulate.

##### Culture characteristics.

Colonies on PDA at room temperature (20–25 °C), covering the whole plate within one week (6 cm diameter Petri dish), white, circular, rough, raised, entire margin; reverse white. Sporulation occurred after prolonged incubation on PDA covered with natural rubber discs.

##### Material examined.

China, • Yunnan Province, Lincang City, contaminated rubber latex, 28 July 2022, Rui-Fang Xu, L-25 (GMB-W1264), living culture GMBCC2520.

##### Notes.

In this study, a multi-gene phylogenetic analysis based on the ITS, LSU, and *TUB2* gene regions revealed that strain GMBCC2520 belongs to *Paracremonium* and forms an independent branch sister to *P. binnewijzendii* (Fig. 7). Pairwise nucleotide comparison of ITS, LSU, and *TUB2* showed that *P. laticis* (GMBCC2520) differs from *P. binnewijzendii* (CBS 143277, ex-type) by 45/531 bp (8.47%, 9 gaps), 15/774 bp (1.94%, 0 gaps), and 33/338 bp (9.76%, 9 gaps), respectively (Jeewon and Hyde 2016). Morphologically, this collection (GMB-W1264) can be distinguished from *P. binnewijzendii* (CBS H-23246, holotype) by its shorter and mostly integrated conidiogenous cells, which are cylindrical to subcylindrical, occasionally swollen at the base, and lack conspicuous collarettes, whereas *P. binnewijzendii* (CBS H-23246, holotype) possesses erect, unbranched, slightly tapering phialides with a visible collarette. Moreover, *P. laticis* (GMB-W1264) produces broader conidia (3–6 μm vs. (1.5–)2.5–3.5(–4.5) μm) that are ellipsoidal to ovoid, smooth-walled, and typically formed singly, while *P. binnewijzendii* forms narrower, ellipsoidal to fusoid conidia in slimy heads at the phialide apices (Crous et al. 2017). Therefore, strain GMBCC2520 (*P. laticis*) is introduced as a new species based on molecular and morphological analyses. The species was isolated from deteriorated rubber latex collected from a container near a rubber tree in Yunnan Province, China, and was associated with detectable surface alterations and oxidative modification signals on natural rubber during laboratory incubation.

### *Schizophyllum* Fr. [as ‘Schizophyllus’], Observ. mycol. (Havniae) 1: 103 (1815)

#### Schizophyllum commune

Taxon classificationFungiAgaricalesSchizophyllaceae

Observ. mycol. (Havniae) 1: 103 (1815) [as ‘ Schizophyllus communis’]

29072FAA-437F-5B9A-8E38-739DE3A2A365

208403

Fig. 12

##### Description.

Colonies on PDA are white, cottony to flossy, and gradually develop a central bulge, but no fruiting bodies are formed. The mycelium is hyaline, septate, granulate, 3–4.5 μm wide, and with clamp connections. Fruiting structures were not observed in either PDA cultures or cultures grown on PDA with natural rubber discs.

**Figure 12.**
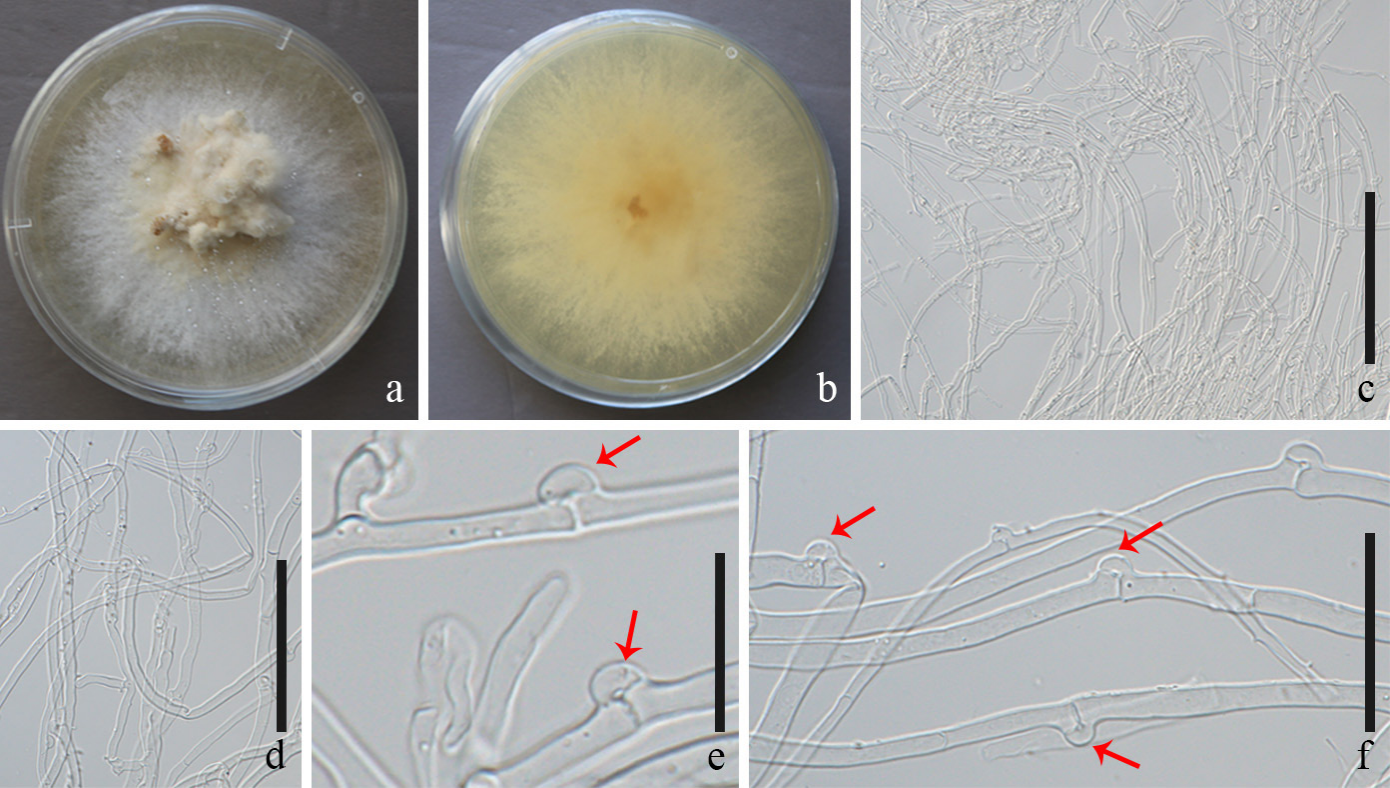
*Schizophyllum commune* (GMB-W1268). **a, b**. Colony on PDA (front and reverse); **c, d**. Mycelium; **e, f**. Clamp connections indicated by arrows. Scale bars: 100 μm (**c, d**), 30 μm (**e, f**).

##### Material examined.

China, • Yunnan Province, Lincang City, contaminated rubber latex, 24 April 2023, Rui-Fang Xu, L-33, (GMB-W1268), living culture GMBCC2521.

##### Culture characteristics.

Colonies on PDA at room temperature (20–25 °C), covering the whole plate within 10 days (6 cm diameter Petri dish), white, cottony to flossy, filamentous, raised, with a filiform margin; reverse yellow to pale yellow.

##### Notes.

Combined multilocus phylogenetic analyses identified strain GMBCC2521 as *Schizophyllum commune* (Fig. 8). Morphological observation showed that the strain (GMB-W1268) did not produce spores. However, clamp connections, which are typical of basidiomycetes, were clearly observed on the hyphae (Fig. 12).

*Schizophyllum commune* is a widely distributed white-rot fungus prevalent in tropical and subtropical regions (Robledo et al. 2014). It is recognized for its ligninolytic enzyme system and its ability to degrade structurally complex plant polymers (Kumar et al. 2022). Previous studies have demonstrated that certain white-rot fungi, including *Phlebia radiata*, *Pleurotus ostreatus*, *Resinicium bicolor*, and *Trametes versicolor*, are capable of degrading rubber (Bredberg et al. 2002; Singh et al. 2017; Andler et al. 2021; Puello-Mendez et al. 2022).

Strain GMBCC2521 was isolated from deteriorated natural rubber latex collected in Yunnan Province, China. During laboratory incubation, the strain was associated with measurable mass-loss trends and observable surface-associated changes on natural rubber discs. Although the ability of white-rot fungi to modify complex polymeric materials has been widely reported, the present observations suggest that *Schizophyllum commune* may be associated with early-stage surface deterioration and oxidative modification of natural rubber under the experimental conditions.

## Discussion

Four fungal species, *Paracremonium laticis* sp. nov., *Neocosmospora bostrycoides*, *Neocosmospora* sp., and *Schizophyllum commune*, were identified from Yunnan Province, China, and Chiang Rai Province, Thailand, as fungi associated with early-stage surface alteration of natural rubber. To date, 15,266 new fungal species have been documented across all 34 provincial-level regions of China. Yunnan Province has reported the highest number of species, with 3,869 species, accounting for nearly one-fifth of the national total (Wang et al. 2021). The southwestern region of China demonstrates the greatest diversity of newly described fungal species (Chia et al. 2025; Du et al. 2025; Han et al. 2025, 2026; Lu et al. 2025a, 2025b; Wanasinghe et al. 2025; Zheng et al. 2026), likely due to its mountainous terrain, extensive vegetation, temperate climate, and abundant rainfall.

In the rubber-degrading experiments, the rubber weight decreased by 0.77% ± 0.27%, 0.82% ± 0.29%, 1.03% ± 0.14%, and 1.24% ± 0.25% after 2 months of growth for strains L-25, L-33, L-12A, and T-21, respectively. Positive Schiff’s reagent staining suggested the possible presence of oxidative surface-associated products. Furthermore, SEM revealed cracks, erosion-like features, and irregular surface structures, consistent with fungal-associated surface deterioration during incubation. Collectively, these observations suggest that the tested fungi were associated with oxidative and structural surface modifications.

However, these observations require cautious interpretation. The relatively low mass-loss values observed in this study, combined with the SEM and Schiff’s reagent results, primarily indicate surface-level modification and oxidative deterioration rather than extensive degradation of the polymer matrix. Consequently, the present results offer preliminary evidence that these fungi are involved in early-stage surface deterioration of natural rubber rather than complete biodegradation.

Traditionally, weight loss has been used as the primary indicator of polymer biodegradation (Nayanashree and Thippeswamy 2015a, 2015b; Basik et al. 2021a). In this study, all four isolates showed a measurable decrease in rubber weight, indicating partial utilization of the substrate. However, the limitations of this method also became apparent, as fungal mycelia adhered firmly to the rubber discs and were not always completely removed during washing. In some cases, the residual mycelium increased the disc weight, making interpretation difficult. Therefore, mass loss alone is not a reliable method for determining whether direct polymer degradation occurs.

Compared with previously reported rubber-degrading microorganisms (Table 5), the surface-associated mass-loss trends observed in this study (0.77–1.24% after 60 days) were relatively low. Prior research has documented substantially higher degradation efficiencies for several bacteria and fungi, including *Nocardia* sp. (10–55.3%), *Bacillus subtilis* (48.6%), *Streptomyces labedae* (41.4%), *Aspergillus oryzae* (57%), and *Ceriporiopsis subvermispora* (35.4%) under varying experimental conditions and incubation periods. Degradation efficiency is influenced by factors such as substrate composition, incubation conditions, microbial physiology, and analytical methodologies. Many previous studies utilized additional analytical techniques, including Fourier transform infrared spectroscopy (FTIR), gas chromatography–mass spectrometry (GC–MS), X-ray photoelectron spectroscopy (XPS), nuclear magnetic resonance spectroscopy (NMR), and mineralization-associated assays, which may provide stronger evidence for polymer modification and chain cleavage. In contrast, the present study primarily demonstrated limited mass loss, SEM-observed surface alteration, and positive Schiff’s reagent staining. Consequently, these findings should be regarded as indicative of oxidative surface modification and early-stage deterioration of natural rubber rather than extensive breakdown of the polymer matrix.

**Table 5.** Comparative analysis of natural rubber degradation by microorganisms with methodological categories.

Microorganism	Type	Substrate	Incubation time	Reported mass loss/modification efficiency (%)	Key analytical methods	References
*Rhodococcus rhodochrous*	Bacterium	Vulcanized rubber particles	14 days	19.32%	Weight loss, SEM, FTIR, enzyme assay	Andler et al. 2022
*Nocardia* sp.	Bacterium	Natural and synthetic rubber	6–8 weeks	10–55.3%	Weight loss, SEM, FTIR	Bode et al. 2001; Sarkar et al. 2022
*Bacillus subtilis*	Bacterium	Natural rubber	2 months	48.60%	Weight loss, SEM, FTIR, enzyme assay	Nayanashree and Thippeswamy 2015a
*Streptomyces labedae*	Bacterium	Natural rubber	4 weeks	41.40%	Molecular weight reduction, staining, SEM	Hesham et al. 2015
*Aspergillus oryzae*	Fungus	Natural rubber	7 months	57%	Weight loss, SEM, FTIR, GC–MS, enzyme assay	El-Wafai et al. 2023
*Ceriporiopsis subvermispora*	Fungus	Vulcanized rubber particles	200 days	35.40%	Weight loss, SEM, FTIR, XPS, NMR	Sato et al. 2004
*Penicillium chrysogenum*	Fungus	Natural rubber	2 months	29.30%	Weight loss, SEM, FTIR, enzyme assay	Nayanashree and Thippeswamy 2015b
*Pleurotus ostreatus*	Fungus	Vulcanized rubber particles	4 weeks	6.10%	Weight loss, SEM, FTIR, enzyme assay	Andler et al. 2021
*Trametes versicolor*	Fungus	Vulcanized rubber particles	4 weeks	7.50%	Weight loss, SEM, FTIR, enzyme assay	Andler et al. 2021

The enzymatic assays performed in this study served as preliminary qualitative screening tests rather than quantitative analyses, with each strain tested only once. Although this approach was appropriate for the initial evaluation of potential enzymatic activities, the absence of biological replication limits the statistical reliability and reproducibility of the results. The observed esterase, lipase, and protease activities may contribute to early-stage surface modification of natural rubber. Laccases may participate in oxidative changes of rubber-associated compounds, whereas esterases, lipases, and proteases may act on additives or oxidized surface materials. However, as direct evidence of polyisoprene chain cleavage was not obtained, the relationship between enzyme production and rubber surface alteration remains unresolved and should be interpreted with caution. Additional biochemical and molecular studies are required to elucidate the mechanisms underlying fungal interactions with natural rubber surfaces.

Previous studies indicate that oxidative enzymes, such as rubber oxygenases, laccases, and peroxidases, participate in microbial interactions with rubber materials (Birke and Jendrossek 2014; Prakash et al. 2024). The positive Schiff’s reagent reactions observed in this study support the occurrence of oxidative surface-associated processes during fungal incubation. However, this study did not obtain direct evidence for polyisoprene chain cleavage, specific degradation products, or enzyme-mediated depolymerization. Additional biochemical and proteomic analyses are needed to elucidate the mechanisms underlying fungal-mediated rubber surface modification.

The present study broadens the taxonomic range of fungi associated with rubber surface deterioration. Previous studies have documented rubber degradation in genera such as *Aspergillus*, *Botryotinia*, *Cladosporium*, *Fusarium*, *Mucor*, *Penicillium*, and *Trichoderma* (Atagana et al. 1999; Ismail et al. 2013; Shah et al. 2013; Nayanashree and Thippeswamy 2015b; Andler et al. 2021; Ibrahim et al. 2024). No direct experimental evidence has demonstrated measurable surface-associated modification of natural rubber by *Neocosmospora*, *Paracremonium*, or *Schizophyllum* prior to this work.

*Neocosmospora* species are widely distributed, occurring as saprobes, plant pathogens, endophytes, and opportunistic human pathogens (Lombard et al. 2015; Sandoval-Denis et al. 2019). Currently, there is no direct evidence that *Neocosmospora* can participate in surface alteration of rubber or plastic materials. Only a few plastic-degradation screening studies have isolated such fungi in plastic-related environments, but no experimental evidence has been provided (Pang et al. 2023). In contrast, the teleomorphs of the *Fusarium solani* species complex (*Fusarium solani*) have been reported to modify or deteriorate polybutylene succinate (PBS), polyurethane (PU), and rubber (Mao et al. 2015; Ren et al. 2021; Joseph et al. 2022).

*Paracremonium* species are widely distributed and have been isolated from diverse substrates worldwide, including soil, water, sewage, humans, trees, and insect associates (Ming et al. 2021; Liu et al. 2024). Their life modes are consequently diverse, encompassing saprobic, pathogenic, and symbiotic relationships (Lombard et al. 2015; Crous et al. 2017; Ming et al. 2021). No rubber or plastic degradation has been reported for *Paracremonium*. This is the first report in this genus showing that *Paracremonium laticis* is associated with surface alteration of natural rubber during incubation.

*Schizophyllum*, belonging to the family *Schizophyllaceae (Agaricales)*, represents one of the most widely distributed groups of basidiomycetous fungi (Siqueira et al. 2016). Members of this genus function as saprobes or pathogens and are commonly found on decaying wood, branches, or tree trunks in natural forests, plantations, and home gardens (Shirokikh and Shirokikh 2024; Kleijburg and Wösten 2025). It has been reported that *Schizophyllum commune* can attack complex synthetic and natural polymers and degrade bis(2-hydroxyethyl) terephthalate (BHET), polyethylene (PE), polyethylene terephthalate (PET), and low-density polyethylene (LDPE) (Perera et al. 2021; Puello-Mendez et al. 2022; Bautista-Zamudio et al. 2023; Martínez et al. 2025). The present finding supports previous observations of the oxidative capabilities of *S. commune* and extends its substrate range to include natural rubber.

Several limitations should be acknowledged in this study. First, only limited surface-associated changes in natural rubber were observed, indicating that the experiments primarily reflected early-stage fungal colonization and surface modification. Second, fungal biomass attached to the rubber surface may have interfered with accurate mass-loss measurements, complicating the distinction between fungal growth and altered rubber. Third, the evidence was restricted to preliminary indications of oxidative surface alteration based on Schiff’s reagent staining and SEM observations and does not confirm extensive polymer degradation or direct polyisoprene chain cleavage. Additionally, the enzyme assays conducted were qualitative and intended solely for preliminary isolate screening. Molecular weight analysis of rubber polymers was not performed, and degradation metabolites were not identified. Despite these limitations, the principal finding is that *Neocosmospora bostrycoides*, *Neocosmospora* sp., *Paracremonium laticis*, and *Schizophyllum commune* are associated with early-stage oxidative surface alteration of natural rubber.

Future research should employ advanced analytical and molecular techniques to elucidate the mechanisms underlying fungal-mediated surface alteration of natural rubber. Methods such as FTIR, GC–MS, Raman spectroscopy, gel permeation chromatography (GPC), and metabolomic profiling could characterize structural changes in the polymer and identify oxidation products formed during fungal incubation. Furthermore, proteomic and enzymatic analyses, including enzyme purification, sodium dodecyl sulfate–polyacrylamide gel electrophoresis (SDS–PAGE), and liquid chromatography–tandem mass spectrometry (LC–MS/MS)-based protein identification, may facilitate the identification of oxidative enzymes involved in rubber transformation. Whole-genome sequencing and comparative genomic analyses could also identify genes associated with oxidative pathways, such as putative rubber oxygenases and other extracellular enzymes implicated in polymer modification. Collectively, these approaches would provide more robust mechanistic evidence for fungal interactions with natural rubber and support the evaluation of their potential applications in sustainable rubber waste management.

Rubber-degrading bacteria have been isolated from diverse environments, including soil, sewage from deteriorated vehicle tires, and latex-contaminated soil. These bacteria are capable of degrading various rubber substrates, such as natural rubber, synthetic rubber, vulcanized rubber, and rubber tires (Cui et al. 2023). Genera recognized for their biodegradative capacity include *Achromobacter* (Berekaa et al. 2005), *Acinetobacter* (Bode et al. 2001), *Actinomadura* (Ibrahim et al. 2006), *Actinoplanes* (Imai et al. 2011), *Amycolatopsis* (Heisey and Spiro 1995), *Bacillus* (Cherian and Jayachandran 2009), *Escherichia* (Guajardo-Flores et al. 2025), *Gordonia* (Linos et al. 2000), *Methylibium* (Imai et al. 2011), *Micromonospora* (Linos et al. 2000), *Mycobacterium* (Berekaa et al. 2000), *Nocardia* (Ibrahim et al. 2006), *Nocardioides* (Gibson et al. 2020), *Nitratireductor* (Itokazu et al. 2025), *Nonomuraea* (Basik et al. 2021b), *Paenibacillus* (Hapuarachchi et al. 2016), *Pseudomonas* (Roy et al. 2006), *Rhizobacter* (Imai et al. 2011), *Rhodococcus* (Watcharakul et al. 2016), *Steroidobacter* (Shah et al. 2013), *Streptomyces* (Hesham et al. 2015), and *Xanthomonas* (Bode et al. 2001). Notably, *Steroidobacter* demonstrated exceptional degradation efficiency in natural rubber decomposition experiments, achieving up to 60% mass loss within 7 days of incubation (Joseph et al. 2022). Investigating the combined application of fungi and bacteria is a promising area of research, as their synergistic activity may accelerate rubber degradation and contribute to sustainable waste management solutions (Cui et al. 2023).

*Neocosmospora bostrycoides*, *Neocosmospora* sp., *Paracremonium laticis* sp. nov., and *Schizophyllum commune* were associated with oxidative surface-associated modification and early-stage deterioration of natural rubber under laboratory incubation conditions. These observations expand current knowledge of fungal diversity associated with deteriorated rubber materials and provide a preliminary foundation for future studies investigating fungal-mediated interactions with natural rubber surfaces.

## Supplementary Material

XML Treatment for Neocosmospora bostrycoides

XML Treatment for Neocosmospora

XML Treatment for Paracremonium laticis

XML Treatment for Schizophyllum commune

## Additional information

### Artificial Intelligence (AI) use

The authors accept full responsibility for the content of the manuscript, including the disclosure of any use of AI.

No AI tools were used in the preparation of this manuscript.

### Author ORCIDs

R.-F. Xu https://orcid.org/0000-0003-1207-8254

S.C. Karunarathna https://orcid.org/0000-0001-7080-0781

K.D. Hyde https://orcid.org/0000-0002-2191-0762

C. Phukhamsakda https://orcid.org/0000-0002-1033-937X

S. Rapior https://orcid.org/0000-0002-9105-1561

D.-Q. Dai https://orcid.org/0000-0001-8935-8807

M.-M. Wang https://orcid.org/0000-0003-3747-5432

S. Ali https://orcid.org/0000-0002-3230-1436

J.-Y. Yang https://orcid.org/0009-0000-0375-990X

P. Kakumyan https://orcid.org/0000-0001-8281-1082

Saowaluck Tibpromma https://orcid.org/0000-0002-4706-6547

### Data availability

All of the data that support the findings of this study are available in the main text or Supplementary Information.

## Supplementary materials

Supplementary material 1 Supplementary tables Data type fileEXP Explanation note **table SS1**. Evolution of natural rubber weight after 2 months of incubation with different fungal strains. **table SS2**. GenBank accession numbers for sequences of *Neocosmospora* species and related taxa used in this study. **table SS3**. GenBank accession numbers of *Paracremonium* and related taxa used in this study. **table SS4**. GenBank accession numbers of *Schizophyllum* strains and related taxa used in this stud. https://binary.pensoft.net/file/1681604This dataset is made available under the Open Database License (http://opendatacommons.org/licenses/odbl/1.0/). The Open Database License (ODbL) is a license agreement intended to allow users to freely share, modify, and use this Dataset while maintaining this same freedom for others, provided that the original source and author(s) are credited. AuthorsNames
