# Supplementary material for: Screening and identification of fungal species associated with early-stage deterioration of natural rubber
Source: MycoKeys. 2026 Jun 16;134:119–56. doi: 10.3897/mycokeys.134.195079 (PMC13291771; doi:10.3897/mycokeys.134.195079)
Supplement: Supplementary material 1 — Supplementary tables [file mycokeys-134-119-s001.doc]

**Table S1.** Evolution of natural rubber weight after 2 months of incubation with different fungal strains. No significant mass loss was observed, and some samples showed variable or increased weights among replicates.

| **Strains no.** | **Initial weight *M1* (g)** | **Final weight *M2* (g)** | **Weight loss percentage (*M*, 100%) (notes)** |
| --- | --- | --- | --- |
| 1-1C | 5.77 | 5.74 | 0.52 |
|  | 5.77 | 5.87 | Weight increase |
|  | 5.78 | 5.87 | Weight increase |
| 5-1 | 3.79 | 3.75 | 1.06 |
|  | 3.79 | 3.80 | Weight increase |
|  | 3.81 | 3.81 | 0 |
| G4 | 3.50 | 3.51 | Weight increase |
|  | 3.52 | 3.47 | 1.42 |
|  | 3.53 | 3.53 | Weight increase |
| L-11A | 4.24 | 4.24 | 0 |
|  | 4.25 | 4.28 | Weight increase |
|  | 4.26 | 4.26 | 0 |
| L-12B | 3.98 | 3.96 | 0.50 |
|  | 4.00 | 3.97 | 0.75 |
|  | 4.04 | 4.05 | Weight increase |
| L-13 | 4.63 | 4.63 | 0 |
|  | 4.63 | 4.63 | 0 |
|  | 4.63 | 4.63 | 0 |
| L-17 | 4.47 | 4.47 | 0 |
|  | 4.47 | 4.45 | 0.45 |
|  | 4.49 | 4.47 | 0.67 |
| L-23B | 4.28 | 4.26 | 0.47 |
|  | 4.28 | 4.28 | 0 |
|  | 4.29 | 4.29 | 0 |
| L-24 | 3.67 | 3.67 | 0 |
|  | 3.68 | 3.68 | 0 |
|  | 3.70 | 3.70 | 0 |
| L-28 | 5.11 | 5.10 | 0.20 |
|  | 5.13 | 5.13 | 0 |
|  | 5.15 | 5.13 | 0.39 |
| L-34 | 4.45 | 4.42 | 0.67 |
|  | 4.45 | 4.45 | 0 |
|  | 4.45 | 4.49 | Weight increase |
| L-35 | 4.68 | 4.68 | 0 |
|  | 4.68 | 4.68 | 0 |
|  | 4.68 | 4.67 | 0.21 |
| L-36 | 4.08 | 4.08 | 0 |
|  | 4.08 | 4.08 | 0 |
|  | 4.08 | 4.04 | 0.98 |
| L-37B | 5.15 | 5.15 | 0 |
|  | 5.21 | 5.18 | 0.58 |
|  | 5.23 | 5.24 | Weight increase |
| L-38 | 5.56 | 5.50 | 1.08% |
|  | 5.57 | 5.58 | Weight increase |
|  | 5.61 | 5.61 | 0 |
| X-01 | 5.22 | 5.22 | Weight increase |
|  | 5.27 | 5.30 | Weight increase |
|  | 5.27 | 5.26 | 0.19 |
| X-07 | 4.74 | 4.85 | Weight increase |
|  | 4.74 | 4.86 | Weight increase |
|  | 4.74 | 4.80 | Weight increase |
| X-10-2 | 3.95 | 3.96 | Weight increase |
|  | 3.95 | 3.96 | Weight increase |
|  | 3.95 | 3.93 | 0.51% |
| T2 | 4.14 | 4.14 | 0 |
|  | 4.14 | 4.15 | Weight increase |
|  | 4.14 | 4.17 | Weight increase |
| T5 | 4.53 | 4.52 | 0.22 |
|  | 4.53 | 4.53 | 0 |
|  | 4.53 | 4.53 | 0 |
| T6 | 4.93 | 4.97 | Weight increase |
|  | 4.94 | 4.94 | 0 |
|  | 4.94 | 4.94 | 0 |
| T8 | 4.36 | 4.37 | Weight increase |
|  | 4.36 | 4.38 | Weight increase |
|  | 4.37 | 4.37 | 0 |
| T9 | 5.46 | 5.46 | 0 |
|  | 5.47 | 5.44 | 0.55 |
|  | 5.50 | 5.50 | 0 |
| T10 | 4.95 | 4.99 | Weight increase |
|  | 4.98 | 4.98 | 0 |
|  | 4.98 | 4.99 | Weight increase |
| T15 | 4.75 | 4.77 | Weight increase |
|  | 4.76 | 4.79 | Weight increase |
|  | 4.80 | 4.85 | Weight increase |
| T16-2 | 3.82 | 3.83 | Weight increase |
|  | 3.83 | 3.83 | 0 |
|  | 3.83 | 3.86 | Weight increase |
| T24 | 3.89 | 3.89 | 0 |
|  | 3.89 | 3.90 | Weight increase |
|  | 3.90 | 3.88 | 0.51 |
| T25 | 4.40 | 4.37 | 0.68 |
|  | 4.40 | 4.40 | 0 |
|  | 4.42 | 4.40 | 0.45 |
| T26 | 4.70 | 4.69 | 0.21 |
|  | 4.70 | 4.73 | Weight increase |
|  | 4.70 | 4.71 | Weight increase |
| T29 | 5.02 | 5.04 | Weight increase |
|  | 5.02 | 5.02 | 0 |
|  | 5.02 | 5.07 | Weight increase |
| T32 | 4.16 | 4.20 | Weight increase |
|  | 4.16 | 4.15 | 0.24 |
|  | 4.16 | 4.16 | 0 |
| T36 | 5.84 | 5.83 | 0.17 |
|  | 5.90 | 5.90 | 0 |
|  | 6.02 | 6.01 | 0.17 |

**Table S2.** GenBank accession numbers for sequences of *Neocosmospora* species and related taxa used in this study.

| **Species** | **Strains** | ***tef*1-α** | **ITS** | **LSU** | ***rpb*2** | **References** |
| --- | --- | --- | --- | --- | --- | --- |
| *Fusarium caeruleum* | CBS 113.23 = NRRL 20643 | LR583588 | LR583695 | LR583695 | - | Sandoval-Denis et al. 2019 |
| *Fusarium caeruleum* | CBS 133.73 = NRRL 22286 = ATCC 24389 = IMI 163397 | LR583589 | LR583696 | LR583906 | - | Sandoval-Denis et al. 2019 |
| *Fusarium caucasicum* | CBS 179.35 = NRRL 13954 = IFO 5679 | LR583591 | LR583698 | LR583698 | - | Sandoval-Denis et al. 2019 |
| *Geejayessia atrofusca* | NRRL 22316 | AF178361 | AF178423 | AF178392 | EU329502 | O’Donnell 2000 |
| *Geejayessia cicatricum* | CBS 125552 | HM626644 | HQ728145 | - | HQ728153 | Schroers et al. 2011 |
| *Neocosmospora acutispora* | CBS 145467 T = NRRL 22574 = BBA 62213 | LR583593 | LR583700 | LR583908 | LR583814 | Sandoval-Denis et al. 2019 |
| *Neocosmospora ambrosia* | CBS 571.94 T = NRRL 22346 = BBA 65390 = MAFF 246287 (ex-epitype of *Monacrosporium ambrosium*) | FJ240350 | EU329669 | EU329669 | EU329503 | O’Donnell et al. 2008 |
| *Neocosmospora ambrosia* | NRRL 20438 = IMI 296597 = MAFF 246291 | AF178332 | AF178397 | DQ236357 | JX171584 | O’Donnell 2000 |
| *Neocosmospora ampla* | CBS 202.32 T = BBA 4170 | LR583594 | LR583701 | LR583909 | LR583815 | Sandoval-Denis et al. 2019 |
| *Neocosmospora batatas* | CBS 144397 = NRRL 22400 = BBA 64683 | AF178343 | AF178407 | AF178376 | EU329509 | O’Donnell 2000 |
| *Neocosmospora batatas* | CBS 144398 T = NRRL 22402 = BBA 64954 = FRC S-0567 | AF178344 | AF178408 | AF178377 | FJ240381 | O’Donnell 2000 |
| *Neocosmospora borneensis* | CBS 145462 = NRRL 22579 = BBA 65095 = GJS 85-197 | AF178352 | AF178415 | AF178384 | EU329515 | O’Donnell 2000 |
| *Neocosmospora bostrycoides* | CBS 239.39 = NRRL 22656 | LR583595 | LR583702 | LR583910 | LR583816 | Sandoval-Denis et al. 2019 |
| *Neocosmospora bostrycoides* | CBS 102824 | LR583596 | LR583703 | LR583911 | LR583817 | Sandoval-Denis et al. 2019 |
| *Neocosmospora bostrycoides* | CBS 130328 = NRRL 31169 | DQ246923 | DQ094396 | DQ236438 | EU329564 | Zhang et al. 2006 |
| *Neocosmospora bostrycoides* | CBS 130391 = NRRL 49707 = FMR 8030 | HM347127 | EU329716 | EU329716 | EU329665 | O’Donnell et al. 2008 |
| *Neocosmospora bostrycoides* | CBS 144.25 NT (ex-neotype of *Fusarium bostrycoides*) | LR583597 | LR583704 | LR583912 | LR583818 | Sandoval-Denis et al. 2019 |
| *Neocosmospora bostrycoides* | CBS 392.66 = NRRL 25325 = BBA 69595 | LR583598 | LR583705 | LR583913 | LR583819 | Sandoval-Denis et al. 2019 |
| *Neocosmospora bostrycoides* | NRRL 52701 = ARSEF 6602 | JF740784 | JF740906 | JF740906 | JF741110 | O’Donnell et al. 2012 |
| *Neocosmospora bostrycoides* | GMBCC2519 | - | PZ341930 | PZ341928 | PZ363915 | This study |
| *Neocosmospora brevicona* | CBS 203.31 = NRRL 22234 = BBA 2019 | LR583599 | LR583706 | LR583914 | LR583820 | Sandoval-Denis et al. 2019 |
| *Neocosmospora brevicona* | CBS 204.31 T = NRRL 22659 = BBA 2123 (ex-type of *Hypomyces haematococcus* var. *breviconus*) | LR583600 | LR583707 | LR583915 | LR583821 | Sandoval-Denis et al. 2019 |
| *Neocosmospora brevis* | CBS 130326 = NRRL 28009 = CDC B-5543 | DQ246869 | DQ094351 | DQ236393 | EF470136 | Zhang et al. 2006 |
| *Neocosmospora brevis* | CBS 144387 T = MUCL 16108 | LR583601 | LR583708 | LR583916 | LR583822 | Sandoval-Denis et al. 2019 |
| *Neocosmospora catenata* | CBS 142228 = NRRL 54992 = UTHSC 09–1008 | KC808213 | KC808255 | KC808255 | KC808354 | Kasson et al. 2013 |
| *Neocosmospora catenata* | CBS 142229 T = NRRL 54993 = UTHSC 09–1009 | KC808214 | KC808256 | KC808256 | KC808355 | Kasson et al. 2013 |
| *Neocosmospora crassa* | CBS 144386 T = MUCL 11420 | LR583604 | LR583709 | LR583917 | LR583823 | Sandoval-Denis et al. 2019 |
| *Neocosmospora crassa* | NRRL 46596 | GU170627 | GU170647 | GU170647 | GU170592 | Migheli et al. 2010 |
| *Neocosmospora cryptoseptata* | CBS 145463 T = NRRL 22412 = BBA 65024 | AF178351 | AF178414 | AF178383 | EU329510 | O’Donnell 2000 |
| *Neocosmospora cucurbitae* | CBS 410.62 = NRRL 22658 = CECT 2864 | DQ247640 | LR583710 | LR583918 | LR583824 | Sandoval-Denis et al. 2019 |
| *Neocosmospora cucurbitae* | CBS 616.66 T = NRRL 22399 = BBA 64411 | DQ247592 | LR583711 | LR583919 | LR583825 | Sandoval-Denis et al. 2019 |
| *Neocosmospora cyanescens* | CBS 518.82 T | LR583605 | AB190389 | LR583920 | LR583826 | Sandoval-Denis et al. 2019 |
| *Neocosmospora cyanescens* | CBS 637.82 | LR583606 | LR583712 | LR583921 | LR583827 | Sandoval-Denis et al. 2019 |
| *Neocosmospora diminuta* | CBS 144390 = MUCL 18798 T | LR583607 | LR583713 | LR583922 | LR583828 | Sandoval-Denis et al. 2019 |
| *Neocosmospora elegans* | CBS 144395 = NRRL 22163 = MAFF 238540 = ATCC 18690 | AF178328 | AF178394 | AF178363 | EU329496 | O’Donnell 2000 |
| *Neocosmospora elegans* | CBS 144396 ET = NRRL 22277 = MAFF 238541 = ATCC 42366 (ex-epitype of *Nectria elegans*) | AF178336 | AF178401 | AF178370 | FJ240380 | O’Donnell 2000 |
| *Neocosmospora euwallaceae* | CBS 135854T = NRRL 54722 (ex-type of *Fusarium euwallaceae*) | JQ038007 | JQ038014 | JQ038014 | JQ038028 | Freeman et al. 2013 |
| *Neocosmospora euwallaceae* | NRRL 62626 | KC691532 | KC691560 | KC691560 | KU171702 | Kasson et al. 2013 |
| *Neocosmospora falciformis* | CBS 475.67 T = IMI 268681 | LT906669 | MG189935 | MG189915 | LT960558 | Sandoval-Denis et al. 2019 |
| *Neocosmospora falciformis* | CBS 121450 | JX435161 | JX435211 | JX435211 | JX435261 | Debourgogne et al. 2012 |
| *Neocosmospora falciformis* | CBS 141593 T = CML 1830 (ex-type of *Fusarium paranaense*) | KF597797 | MG787463 | MG787463 | KF680011 | Sandoval-Denis et al. 2019 |
| *Neocosmospora ferruginea* | CBS 109028 T = NRRL 32437 | DQ246979 | DQ094446 | DQ236488 | EU329581 | Zhang et al. 2006 |
| *Neocosmospora ferruginea* | CPC 28194 | LR583602 | LT746276 | LT746276 | LT746341 | Sandoval-Denis et al. 2019 |
| *Neocosmospora gamsii* | CBS 143207 T = NRRL 32323 = UTHSC 99–205 | DQ246951 | DQ094420 | DQ236462 | EU329576 | Zhang et al. 2006 |
| *Neocosmospora gamsii* | CBS 143209 = NRRL 32770 = FRC S–0524 | DQ247083 | DQ094544 | DQ236586 | EU329615 | Zhang et al. 2006 |
| *Neocosmospora guarapiensis* | CBS 131752 = GJS 93–44 | LR583608 | LR583714 | LR583714 | LR583829 | Sandoval-Denis et al. 2019 |
| *Neocosmospora haematococca* | CBS 119600 ET = FRC S–1832 (ex-epitype of *Nectria haematococca*) | DQ247510 | KM231797 | KM231664 | LT960561 | Sandoval-Denis et al. 2019 |
| *Neocosmospora henyangensis* | HMAS 254518 T | KY829448 | KY829446 | - | - | Zeng and Zhuang 2017 |
| *Neocosmospora hypothenemi* | CBS 145464 T = NRRL 52782 = ARSEF 5878 | JF740850 | LR583715 | LR583923 | JF741176 | O’Donnell et al. 2012 |
| *Neocosmospora hypothenemi* | CBS 145466 = NRRL 52783 = ARSEF 5879 | JF740851 | LR597067 | LR597068 | JF741177 | O’Donnell et al. 2012 |
| *Neocosmospora illudens* | NRRL 22090 = BBA 67606 = GJS 82–98 | AF178326 | AF178393 | AF178362 | JX171601 | O’Donnell 2000 |
| *Neocosmospora ipomoeae* | CBS 225.58 = NRRL 22235 = BBA 64431 | LR583609 | LR583716 | LR583924 | LR583830 | Sandoval-Denis et al. 2019 |
| *Neocosmospora ipomoeae* | CBS 353.87 = NRRL 22657 | DQ247639 | LR583717 | LR583925 | LR583831 | Sandoval-Denis et al. 2019 |
| *Neocosmospora keleraja* | CBS 125720 PT = FRC S–1837 = GJS 02–114 | LR583612 | LR583720 | LR583928 | LR583834 | Sandoval-Denis et al. 2019 |
| *Neocosmospora keleraja* | CBS 125722 PT = FRC S–1836 = GJS 02–114 | DQ247515 | JF433039 | JF433039 | LR583835 | Sandoval-Denis et al. 2019 |
| *Neocosmospora keratoplastica* | CBS 490.63 T (ex-type of *Cephalosporium keratoplasticum*) | LT906670 | MH858333 | NG_088073 | LT960562 | Sandoval-Denis et al. 2019 |
| *Neocosmospora keratoplastica* | FRC S–2477 T (ex-type of *Fusarium keratoplasticum*) | JN235712 | NR_130690 | JN235282 | - | Short et al. 2011 |
| *Neocosmospora kuroshio* | CBS 142642 T | KX262216 | LR583723 | LR583931 | LR583837 | Sandoval-Denis et al. 2019 |
| *Neocosmospora kuroshio* | NRRL 62945 | KM406629 | KM406636 | KM406636 | KM406649 | O’Donnell et al. 2015 |
| *Neocosmospora kurunegalenis* | CBS 119939 T = GJS 02-94 | DQ247511 | JF433036 | JF433036 | LR583838 | O’Donnell et al. 2012 |
| *Neocosmospora lichenicola* | CBS 166.67 T = IMUR 1797 (ex-type of *Moeszia pernambucensis*) | LR583614 | LR583724 | LR583932 | - | Sandoval-Denis et al. 2019 |
| *Neocosmospora lichenicola* | CBS 279.34 T (ex-type of *Monacrosporium tedeschi*) | LR583615 | LR583725 | LR583933 | LR583840 | Sandoval-Denis et al. 2019 |
| *Neocosmospora lichenicola* | CBS 279.59 T = ATCC 13427 (type of *Mastigosporium heterosporium*) | LR583616 | LR583726 | LR583934 | LR583841 | Sandoval-Denis et al. 2019 |
| *Neocosmospora linodendri* | CBS 117481 T = NRRL 22389 = BBA 67587 = GJS 91–148 | AF178340 | AF178404 | AF178373 | EU329506 | O’Donnell 2000 |
| *Neocosmospora longissima* | CBS 126407 T = GJS 85–72 | LR583621 | LR583731 | LR583939 | LR583846 | Sandoval-Denis et al. 2019 |
| *Neocosmospora macrospora* | CBS 142424 = CPC 28191 | LT746218 | LT746266 | LT746281 | LT746331 | Sandoval-Denis et al. 2019 |
| *Neocosmospora macrospora* | CPC 28193 | LT746220 | LT746268 | LT746283 | LT746333 | Sandoval-Denis et al. 2019 |
| *Neocosmospora mahasenii* | CBS 119594 T | DQ247513 | JF433045 | JF433045 | LT960563 | Zhang et al. 2006 |
| *Neocosmospora mahasenii* | FRC S-1840 = GJS 02-124 | DQ247520 | JF433042 | JF433042 | - | Zhang et al. 2006 |
| *Neocosmospora martii* | BPI 452385 LT (lectotype of *Fusarium martii*) | LR583625 | LR583735 | LR583943 | - | Sandoval-Denis et al. 2019 |
| *Neocosmospora martii* | CBS 115659 ET = FRC S-0679 = MRC 2198 (ex-epitype of *Fusarium martii*) | JX435156 | JX435206 | JX435206 | JX435256 | Debourgogne et al. 2012 |
| *Neocosmospora martii* | CBS 142423 T (ex-type of *Neocosmospora croci*) | LT746216 | LT746264 | - | LT746329 | Sandoval-Denis et al. 2019 |
| *Neocosmospora metavorans* | CBS 233.36 = NRRL 22654 | DQ247636 | LR583737 | LR583945 | LR583848 | Sandoval-Denis et al. 2019 |
| *Neocosmospora metavorans* | CBS 135789 T | LR583627 | LR583738 | LR583946 | LR583849 | Sandoval-Denis et al. 2019 |
| *Neocosmospora mori* | CBS 145467 T = NRRL 22230 = MAFF 238539 | AF178358 | DQ094305 | DQ236347 | EU329499 | O’Donnell et al. 2008 |
| *Neocosmospora mori* | CBS 145468 = NRRL 22157 = MAFF 238538 | AF178359 | DQ094306 | DQ236348 | EU329493 | O’Donnell et al. 2008 |
| *Neocosmospora nirenbergiana* | CBS 145469 T = NRRL 22387 = BBA 65023 = GJS 87–127 | AF178339 | AF178403 | AF178372 | EU329505 | O’Donnell 2000 |
| *Neocosmospora noneumartii* | CBS 115658 T = FRC S–0661 | LR583630 | LR583745 | LR583949 | LR583852 | Sandoval-Denis et al. 2019 |
| *Neocosmospora noneumartii* | Fs112 | DQ164848 | DQ164844 | - | - | Zhang et al. 2006 |
| *Neocosmospora oblonga* | CBS 130325 T = NRRL 28008 = CDC B–4701 | LR583631 | LR583746 | LR583950 | LR583853 | Sandoval-Denis et al. 2019 |
| *Neocosmospora oligoesplalta* | CBS 143241 T = NRRL 62579 = FRC S-2581 = MAFF 246283 (ex-type of *Fusarium oligoseptatum*) | KC691538 | KC691566 | KC691566 | LR583854 | Kasson et al. 2013 |
| *Neocosmospora oligoesplalta* | NRRL 62578 = FRC S–2576 | KC691537 | KC691565 | KC691565 | KC691626 | Kasson et al. 2013 |
| *Neocosmospora paraeumartii* | CBS 487.76 T = NRRL 13997 = BBA 62215 | DQ247549 | LR583747 | LR583951 | LR583855 | Sandoval-Denis et al. 2019 |
| *Neocosmospora parceramosa* | CBS 115695 T = CPC 1246 = STE-U 1246 | JX435149 | JX435199 | JX435199 | JX435249 | Debourgogne et al. 2012 |
| *Neocosmospora parceramosa* | NRRL 31158 | DQ246916 | DQ094389 | DQ236431 | EU329559 | Zhang et al. 2006 |
| *Neocosmospora parva* | CBS 466.70 IT = ATCC 26343 | LR583632 | LR583748 | LR583382 | LR583886 | Sandoval-Denis et al. 2019 |
| *Neocosmospora perseae* | CBS 144142 T = CPC 26829 | LT991902 | LT991940 | LT991947 | LT991909 | Sandoval-Denis et al. 2019 |
| *Neocosmospora perseae* | CBS 144143 = CPC 26830 | LT991903 | LT991941 | LT991948 | LT991910 | Sandoval-Denis et al. 2019 |
| *Neocosmospora petroliphile* | NRRL 43812 = CDC 2006743705 | EF453054 | EF453205 | EF453205 | EF470093 | O’Donnell et al. 2007 |
| *Neocosmospora petroliphile* | NRRL 46706 = FMR 8340 | - | EU329715 | EU329715 | EU329664 | O’Donnell et al. 2008 |
| *Neocosmospora phaseoli* | NRRL 36877 T= MAFF 239757 (ex-type of *Fusarium aasssipitatum*) | AY320161 | AY320197 | AY320143 | EU329566 | Aoki et al. 2003 |
| *Neocosmospora phaseoli* | NRRL 54364 T=MAFF 242371 (ex-type of *Fusarium azukcoia*) | FJ240351 | FJ240376 | FJ240376 | FJ240405 | O’Donnell et al. 2008 |
| *Neocosmospora piperis* | CBS 145470 T = NRRL 22570 = GJS 89-14 = CML 1888 | AF178360 | AF178422 | AF178391 | EU329513 | O’Donnell 2000 |
| *Neocosmospora pisi* | CBS 123669 T = NRRL45880 = ATCC MYA-4622 = Wnetten 77–13–4 (ex-epitype of *Fusarium marli* var. *pis*) | LR583636 | LR583753 | LR583957 | LR583862 | Sandoval-Denis et al. 2019 |
| *Neocosmospora pisi* | CBS 124896 = IHEM 15469 | JX435130 | JX435180 | JX435180 | JX435230 | Debourgogne et al. 2012 |
| *Neocosmospora plagianthi* | NRRL 22632 = GJS 89–146 | AF178354 | AF178417 | AF178386 | JX171614 | O’Donnell 2000 |
| *Neocosmospora protoensiformis* | CBS 145471 T = NRRL 22178 = GJS 90-168 | AF178334 | AF178399 | AF178368 | EU329498 | O’Donnell 2000 |
| *Neocosmospora pseudensiformis* | CBS 130.78 = NRRL 22575 = NRRL 22653 | MW620184 | MH863652 | MH875116 | MW474709 | Sandoval-Denis et al. 2019 |
| *Neocosmospora pseudoradicicola* | NRRL 25138 = ARSEF 2314 | JF740758 | JF740900 | JF740900 | - | O’Donnell et al. 2012 |
| *Neocosmospora pseudotonkinensis* | CBS 143038 | LR583640 | LR583758 | LR583962 | LR583867 | Sandoval-Denis et al. 2019 |
| *Neocosmospora quercicola* | CBS 141.90 T = NRRL 22652 | DQ247634 | LR583760 | LR583964 | LR583869 | Sandoval-Denis et al. 2019 |
| *Neocosmospora quercicola* | NRRL 32758 = FRC S-0429 | DQ247056 | DQ094517 | DQ236559 | EU329605 | Zhang et al. 2006 |
| *Neocosmospora rectiphora* | CBS 125727 T = GJS 02-89 = FRC S–1831 | DQ247509 | JF433034 | JF433034 | LR583871 | Sandoval-Denis et al. 2019 |
| *Neocosmospora rectiphora* | HMAS 254519 T (ex-type of *Neocosmospora bomiensis*) | KY829449 | KY829447 | - | - | Zeng and Zhuang 2017 |
| *Neocosmospora regularis* | NRRL 22355 = BBA 60075 | AF178342 | AF178406 | AF178375 | EU329508 | O’Donnell 2000 |
| *Neocosmospora regularis* | CBS 190.35 | LR583642 | LR583762 | LR583966 | LR583872 | Sandoval-Denis et al. 2019 |
| *Neocosmospora regularis* | CBS 230.34 T | LR583643 | LR583763 | LR583967 | LR583873 | Sandoval-Denis et al. 2019 |
| *Neocosmospora riograndensis* | UPMG CM F12570 T | KX534002 | KT186366 | KX534001 | KX534003 | Sandoval-Denis et al. 2019 |
| *Neocosmospora robusta* | CBS 145473 T = NRRL 22385 = BBA 65682 | AF178341 | AF178405 | LR583968 | EU329507 | Sandoval-Denis et al. 2019 |
| *Neocosmospora samuelsii* | CBS 114067 T = GJS 89–70 | LR583644 | LR583764 | LR583969 | LR583874 | Sandoval-Denis et al. 2019 |
| *Neocosmospora silvicola* | CBS 123846 T= GJS 04–67 | LR583646 | LR583766 | LR583971 | LR583876 | Sandoval-Denis et al. 2019 |
| *Neocosmospora silvicola* | NRRL 22161 = ATCC 18692 | AF178330 | DQ094311 | DQ236353 | EU329494 | Zhang et al. 2006 |
| *Neocosmospora solani* | BPI 451321 (lectotype of *Fusarium aduncisporum)* | LR583647 | LR583767 | LR583972 | - | Sandoval-Denis et al. 2019 |
| *Neocosmospora solani* | CBS 101018 T (ex-type of *Neocosmospora rubicola*) | LR583651 | LR583770 | LR583975 | LR583878 | Sandoval-Denis et al. 2019 |
| *Neocosmospora solani* | CBS 111772 | LR583652 | LR583771 | LR583976 | LR583879 | Sandoval-Denis et al. 2019 |
| *Neocosmospora solani* | CBS 112101 | LR583653 | LR583772 | LR583977 | LR583880 | Sandoval-Denis et al. 2019 |
| *Neocosmospora solani* | CBS 117149 | LR583654 | LR583773 | LR583978 | LR583881 | Sandoval-Denis et al. 2019 |
| *Neocosmospora solani* | CBS 119996 | JX435152 | JX435202 | JX435202 | JX435252 | Debourgogne et al. 2012 |
| *Neocosmospora spathuiata* | CBS 145474 T = NRRL28541=UTHSC 98–1305 | DQ246982 | EU329674 | EU329674 | EU329542 | O’Donnell et al. 2008 |
| *Neocosmospora sterdicoia* | CBS 142480 T (ex-type of *Fusarium witzenhousenense*) | - | LR583778 | LR583983 | LR583886 | Sandoval-Denis et al. 2019 |
| *Neocosmospora sterdicoia* | CBS 1424811 = DSM 106211(ex-type of *Fusarium serdcoie*) | LR583868 | LR583779 | LR583984 | LR583887 | Sandoval-Denis et al. 2019 |
| *Neocosmospora striata* | CBS 105.77T = NRRL22427 = NRRL22443 = ATCC34720 = IFM4528 = IMI210879 = NHL2745 | DQ247604 | LR583781 | LR583781 | LR583889 | Sandoval-Denis et al. 2019 |
| *Neocosmospora tenuicistata* | IMI 277708 = NHL2911 | - | LR583782 | LR583986 | - | Sandoval-Denis et al. 2019 |
| *Neocosmospora theobromae* | BPI 453072 T | LR583860 | - | LR583987 | - | Sandoval-Denis et al. 2019 |
| *Neocosmospora tonkinensis* | CBS 115.40 T | LT906672 | MG189941 | MG189926 | LT746340 | Sandoval-Denis et al. 2019 |
| *Neocosmospora tonkinensis* | CBS 222.49 | LR583961 | LR583783 | LR583988 | LR583890 | Sandoval-Denis et al. 2019 |
| *Neocosmospora vasinfeda* | CBS 406.82 | - | LR583790 | LR583995 | LR583897 | Sandoval-Denis et al. 2019 |
| *Neocosmospora vasinfeda* | CBS 446.93 T = IMI 316967 = NHL2919 (ex-type of *Neocosmospora boninensis*) | - | LR583791 | LR583996 | LR583898 | Sandoval-Denis et al. 2019 |
| *Neocosmospora* sp. | IBFS07 | JX524768 | FJ200220 | - | - | Bueno et al. 2014 |
| *Neocosmospora* sp. | IBFS08 | JX524769 | FJ200221 | - | - | Bueno et al. 2014 |
| *Neocosmospora* sp. (AF-1) | NRRL 22231 | KC691542 | KC691570 | KC691570 | KC691631 | Kasson et al. 2013 |
| *Neocosmospora* sp. (AF-3) | NRRL 62629 | KC691536 | KC691564 | KC691564 | KC691625 | Kasson et al. 2013 |
| *Neocosmospora* sp. (AF-6) | NRRL 62590 | KC691546 | KC691574 | KC691574 | KC691635 | Kasson et al. 2013 |
| *Neocosmospora* sp. (AF-6) | NRRL 62591 | KC691545 | KC691573 | KC691573 | KC691634 | Kasson et al. 2013 |
| *Neocosmospora* sp. (AF-7) | NRRL 62610 | KC691547 | KC691575 | KC691575 | KC691636 | Kasson et al. 2013 |
| *Neocosmospora* sp. (AF-7) | NRRL 62611 | KC691548 | KC691576 | KC691576 | KC691637 | Kasson et al. 2013 |
| *Neocosmospora* sp. (AF-8) | NRRL 62584 | KC691554 | KC691582 | KC691582 | KC691643 | Kasson et al. 2013 |
| *Neocosmospora* sp. (AF-8) | NRRL 62585 | KC691549 | KC691577 | KC691577 | KC691638 | Kasson et al. 2013 |
| *Neocosmospora* sp. (AF-9) | NRRL 22643 = ATCC 44215 | DQ247628 | KC691583 | KC691583 | KC691644 | Kasson et al. 2013 |
| *Neocosmospora* sp. (AF-9) | NRRL 66088 | KM406625 | KM406632 | KM406632 | KM406646 | Kasson et al. 2013 |
| *Neocosmospora* sp. (AF-10) | NRRL 62941 = IMI 351954 | KM406626 | KM406633 | KM406633 | KM406647 | Kasson et al. 2013 |
| *Neocosmospora* sp. (AF-11) | NRRL 62944 | KM406627 | KM406634 | KM406634 | KM406648 | Kasson et al. 2013 |
| *Neocosmospora* sp. (FSSC 12) | CBS 143203 = NRRL 32309 = UTHSC 00–1608 | DQ246937 | DQ094407 | DQ236449 | EU329571 | Zhang et al. 2006 |
| *Neocosmospora* sp. (FSSC 12) | CBS 143206 = NRRL32317 = UTHSC 99–1886 | DQ246945 | DQ094414 | DQ236456 | EU329575 | Zhang et al. 2006 |
| *Neocosmospora* sp. | MFLUCC 26–0156 | - | PZ327755 | PZ327762 | PZ363916 | This study |

**Table S3.** GenBank accession numbers of *Paracremonium* and related taxa used in this study.

| **Species** | **Strains** | **ITS** | **LSU** | ***TUB*2** | **References** |
| --- | --- | --- | --- | --- | --- |
| *Corallomycetella elegans* | CBS 275.60 | KM231828 | KM231710 | KM232100 | Lombard et al. 2015 |
| *Corallomycetella repens* | CBS 118.84 | KC479755 | KM231709 | KC479784 | Lombard et al. 2015 |
| *Corallomycetella repens* | CBS 358.49 | KC479756 | KM231708 | KC479785 | Lombard et al. 2015 |
| *Cosmospora arxii* | CBS 748.69 T | KM231819 | KM231694 | KM232089 | Lombard et al. 2015 |
| *Cosmospora coccinea* | CBS 341.70 T | HQ897827 | KM231692 | KM232086 | Lombard et al. 2015 |
| *Cosmospora cymosa* | CBS 762.69 T | HQ897828 | KM231693 | KM232087 | Lombard et al. 2015 |
| *Macroconia papilionacearum* | CBS 125495 | HQ897826 | KM231704 | KM232096 | Lombard et al. 2015 |
| *Macroconia leptosphaeriae* | CBS 100001 | HQ897810 | KM231705 | KM232097 | Lombard et al. 2015 |
| *Macroconia leptosphaeriae* | CBS 112770 | KM231826 | KM231706 | KM232098 | Lombard et al. 2015 |
| *Paracremonium apiculatum* | CGMCC3.19309 T | MK329123 | MK329028 | MK336136 | Zhang et al. 2020 |
| *Paracremonium apiculatum* | LC12502 | MK329124 | MK329029 | MK336137 | Zhang et al. 2020 |
| *Paracremonium aquaticum* | MFLUCC 22–0077 T | OP216410 | OP216405 | OP251200 | Zhang et al. 2020 |
| *Paracremonium bendijkiorum* | NL19_24005 T | MW883436 | MW883828 | MW890139 | Crous et al. 2021 |
| *Paracremonium binnewijzendii* | CBS 143277 T | MG250173 | MG250174 | MG254816 | Crous et al. 2017 |
| *Paracremonium binnewijzendii* | MFLUCC 16–1276 | MK828669 | MK828236 | - | Crous et al. 2017 |
| *Paracremonium contagium* | CBS 110348 T | KM231831 | HQ232118 | KM232103 | Lombard et al. 2015 |
| *Paracremonium contagium* | UCR2900 | KP012611 | KP012631 | - | Lynch et al. (2016) |
| *Paracremonium ellipsoideum* | CGMCC3.19316 T | MK329125 | MK329030 | MK336138 | Zhang et al. 2020 |
| *Paracremonium ellipsoideum* | LC12552 | MK329126 | MK329031 | MK336139 | Zhang et al. 2020 |
| *Paracremonium inflatum* | CBS 482.78 | KM231830 | KM231711 | KM232102 | Lombard et al. 2015 |
| *Paracremonium inflatum* | CBS 485.77 T | KM231829 | HQ232113 | KM232101 | Lombard et al. 2015 |
| *Paracremonium laticis* | GMBCC2520 T | PZ341931 | PZ341929 | PZ363914 | This study |
| *Paracremonium moubasheri* | AUMC 11030 T | KX384655 | - | - | Al-Bedak et al. 2019 |
| *Paracremonium pembeum* | UCR2993 T | - | - | KU053066 | Lynch et al. 2016 |
| *Paracremonium pembeum* | UCR2994 | - | - | KU053067 | Lynch et al. 2016 |
| *Paracremonium pembeum* | UCRCFU254 | KP012604 | KP012624 | - | Lynch et al. 2016 |
| *Paracremonium pembeum* | UCRCFU258 | KP012605 | KP012625 | - | Lynch et al. 2016 |
| *Paracremonium* sp. | UCR2359 | KP030842 | KP030846 | KU053052 | Lynch et al. 2016 |
| *Paracremonium* sp. | UCR2323 | KP030840 | KP030844 | KU053050 | Lynch et al. 2016 |
| *Paracremonium* sp. | UCR2330 | KP030841 | KP030845 | KU053051 | Lynch et al. 2016 |
| *Paracremonium* sp. | HKU42 | LC158596 | LC158621 | LC159312 | Lynch et al. 2016 |
| *Paracremonium variiforme* | CGMCC3.17931 T | KU746691 | KU746737 | KU746783 | Zhang et al. 2017 |
| *Paracremonium variiforme* | CGMCC3.17932 | KU746692 | KU746738 | KU746784 | Zhang et al. 2018 |
| *Paracremonium variiforme* | CGMCC3.17933 | KU746693 | KU746739 | KU746785 | Zhang et al. 2019 |
| *Paracremonium yunnanense* | GMBCC1132 T | PV061854 | PV061857 | PX717292 | Liu et al. 2026 |
| *Paracremonium yunnanense* | GMBCC1133 | PV061855 | PV061858 | PX717293 | Liu et al. 2026 |
| *Paracremonium yunnanense* | GMBCC1135 | PV061856 | PV061859 | PX717294 | Liu et al. 2026 |
| *Stachybotrys chartarum* | CBS129.13 OUTGROUP | KM231858 | KM231738 | KM232127 | Lombard et al. 2015 |
| *Varicosporellopsis americana* | CPC 40768 T | OK664740 | OK663779 | OK651212 | Crous et al. 2021 |
| *Varicosporellopsis aquatilis* | CBS 143509 | MH107922 | MH107968 | MH108052 | Lechat and Fournier 2016 |

**Table S4.** GenBank accession numbers of *Schizophyllum* strains and related taxa used in this stud.

| **Species** | **Strain** | **ITS** | **LSU** | ***tef*1-α** | ***rpb*2** | **References** |
| --- | --- | --- | --- | --- | --- | --- |
| *Fistulina hepatica* | F9173 | ON794230 | PP102655 | - | - | GenBank |
| *Gloeophyllum trabeum* | C9.B241 | PX051720 | PV992345 | - | - | GenBank |
| *Lentinus tigrinus* | CIRM-BRFM 745 | PV108013 | - | - | - | GenBank |
| *Panus conchatus* | Dai_25115 | PQ191333 | PQ198088 | - | - | GenBank |
| *Pleurotus ostreatus* | POA | PX956996 | - | - | - | GenBank |
| *Pleurotus pulmonarius* | PDD 124111 | OZ415579 | OZ415548 | - | - | GenBank |
| *Schizophyllum amplum* | FCUG 1803 | AF141873 | AF141873 | - | - | Parmasto and Hallenberg 2000 |
| *Schizophyllum amplum* | NH1803 | DQ097353 | AY293169 | - | - | Binder et al. 2005 |
| *Schizophyllum commune* | CBS 132304 | LT217530 | LT217561 | LT217595 | LT217629 | Siqueira et al. 2016 |
| *Schizophyllum commune* | CBS 476.64 | LT217531 | LT217562 | LT217596 | LT217630 | Siqueira et al. 2016 |
| *Schizophyllum commune* | FMR 14713 | LT217532 | LT217563 | LT217597 | LT217631 | Siqueira et al. 2016 |
| *Schizophyllum commune* | MUCL 29305 | LT217533 | LT217565 | LT217599 | LT217633 | Siqueira et al. 2016 |
| *Schizophyllum commune* | MUCL 30748 | LT217534 | LT217566 | LT217600 | LT217634 | Siqueira et al. 2016 |
| *Schizophyllum commune* | MUCL 31016 | LT217535 | LT217567 | LT217601 | LT217635 | Siqueira et al. 2016 |
| *Schizophyllum commune* | MUCL20578 | - | LT217564 | LT217598 | LT217632 | Siqueira et al. 2016 |
| *Schizophyllum commune* | UTHSCSA DI14–5 | LT217536 | LT217568 | LT217602 | LT217636 | Siqueira et al. 2016 |
| *Schizophyllum commune* | GMBCC2521 | PZ341932 | - | - | - | This study |
| *Schizophyllum fasciatum* | CBS 267.60 | LT217559 | LT217593 | LT217627 | LT217661 | Siqueira et al. 2016 |
| *Schizophyllum leprieurii* | ROBLEDO 1313 | KM098065.1 | KM098066.1 | - | - | Robledo et al. 2014 |
| *Schizophyllum radiatum* | CBS 301.32 | LT217537 | LT217569 | LT217603 | LT217637 | Siqueira et al. 2016 |
| *Schizophyllum radiatum* | UTHSCSA DI14-1 | LT217539 | LT217571 | LT217605 | LT217639 | Siqueira et al. 2016 |
| *Schizophyllum radiatum* | UTHSCSA DI14–2 | LT217540 | LT217572 | LT217606 | LT217640 | Siqueira et al. 2016 |
| *Schizophyllum radiatum* | UTHSCSA DI14–3 | LT217541 | LT217573 | LT217607 | LT217641 | Siqueira et al. 2016 |
| *Schizophyllum radiatum* | UTHSCSA DI14–4 | - | LT217574 | LT217608 | LT217642 | Siqueira et al. 2016 |
| *Schizophyllum radiatum* | UTHSCSA DI14–6 | LT217542 | LT217575 | LT217609 | LT217643 | Siqueira et al. 2016 |
| *Schizophyllum radiatum* | UTHSCSA DI14–7 | - | LT217576 | LT217610 | LT217644 | Siqueira et al. 2016 |
| *Schizophyllum radiatum* | UTHSCSA DI14–8 | LT217543 | LT217577 | LT217611 | LT217645 | Siqueira et al. 2016 |
| *Schizophyllum radiatum* | UTHSCSA DI14–9 | LT217544 | LT217578 | LT217612 | LT217646 | Siqueira et al. 2016 |
| *Schizophyllum* sp. | BAB–4966 | KM051395.1 | - | - | - | GenBank |
| *Schizophyllum* sp. | HM230 | OP829145.1 | - | - | - | Li et al. 2024 |
| *Schizophyllum* cf. *umbrinum* | ROBLEDO 2478 | KM098067 | KM098068 | - | - | Robledo et al. 2014 |
| *Schizophyllum umbrinum* | FL02.1 | AF249391 | AF261590 |  |  | James et al. 2001 |
| *Schizophyllum umbrinum* | MUCL 43017 | LT217560 | LT217594 | LT217628 | LT217662 | Siqueira et al. 2016 |
| *Trametes versicolor* | 212J10 | PX954405 | PX652871 | - | - | GenBank |
